# Optimal dosage of exercise interventions for enhancing inhibitory control in overweight and obese children and adolescents: insights from a multilevel meta-analysis

**DOI:** 10.3389/fpsyg.2026.1870462

**Published:** 2026-06-17

**Authors:** Pengfei Wang, Hongyu Wang, Yu Miao, Jinrong He, Ting Wang, Zhongxu Hu, Zilong Wang, Xianyang Xin, Tiance Jiang, Yongqing Guo, Zhongwei Zhao, Dong Li

**Affiliations:** 1 College of Physical Education and Health, Guangxi Normal University, Guangxi, China; 2 Department of Sports and Leisure, Dongshin University, Naju, Republic of Korea; 3 School of Physical Education, Shanghai University of Sport, Shanghai, China; 4 School of Competitive Sports, Beijing Sport University, Beijing, China; 5 Moray House School of Education and Sport, University of Edinburgh, Edinburgh, United Kingdom; 6 School of Physical Education, Jimei University, Xiamen, China; 7 Capital University of Physical Education and Sports, Beijing, China; 8 Physical Education Institute, Tomsk State University, Tomsk Oblast, Russia; 9 College of Sports Science, Shenyang Normal University, Shenyang, China; 10 School of Physical Education and Health, Zhaoqing University, Zhaoqing, China

**Keywords:** adolescent, children, exercise, inhibitory control, meta-analysis, obesity, overweight

## Abstract

**Background:**

Overweight and obesity in children and adolescents are frequently associated with impairments in inhibitory control. Exercise interventions have been proposed as an effective means to enhance inhibitory control in this population. This study employs a three-level meta-analysis to assess the effects of exercise interventions on inhibitory control in overweight and obese children and adolescents, and further explores the dose–response relationship to identify the optimal intervention dosage.

**Methods:**

Systematic searches were conducted in the PubMed, Web of Science, Embase, Cochrane Library, and CNKI databases to identify relevant studies. Hedges’ g was used as the measure of effect size, and a three-level random-effects model was implemented using the metafor package in R to address the dependency of multiple effect sizes within individual studies.

**Results:**

A total of 11 studies from four countries, involving 787 overweight and obese children and adolescents (average age: 8.75–14.06 years), were included. The meta-analysis revealed that exercise interventions significantly improved inhibitory control in this population, with consistent findings across both the three-level model (*g* = 0.41, *p* = 0.002) and the traditional two-level model (*g* = 0.45, *p* < 0.001). Nonlinear regression analysis using restricted cubic splines showed that the improvement in inhibitory control followed a nonlinear pattern within a specific dosage range, with the optimal intervention being approximately 49 exercise sessions, each lasting 48 min. Additionally, linear meta-regression revealed a significant negative linear relationship between BMI and intervention effect (*β* = −0.13, *p* = 0.027).

**Conclusion:**

Exercise interventions significantly enhance inhibitory control in overweight and obese children and adolescents. Based on current evidence, approximately 49 exercise sessions, each lasting about 48 min, may represent a potentially beneficial intervention dosage for improving inhibitory control. However, given the very low certainty of evidence, these findings should be interpreted cautiously and considered hypothesis-generating rather than definitive clinical recommendations.

**Systematic review registration:**

PROSPERO, CRD420251163768.

## 1 Introduction

In recent years, overweight and obesity among children and adolescents have become a global public health crisis. According to a study published in The Lancet, the combined prevalence of overweight and obesity in this population doubled between 1990 and 2021, while the prevalence of obesity alone tripled (Kerr et al., 1990). Furthermore, data from JAMA Pediatrics indicates that, as of 2023, the obesity rate in children and adolescents is approximately 8.5%, while the rate of overweight is about 14.8% (Zhang et al., 2024). This means that nearly one in five children or adolescents faces the risk of being overweight. Projections suggest that by 2050, the obesity rate in children aged 5 to 14 will rise to 15.6%, with an estimated 186 million affected individuals, posing a long-term and significant challenge to global public health systems (Kerr et al., 1990). In addition to increasing the risk of diabetes, cardiovascular diseases, and metabolic syndrome (Sharma et al., 2019), overweight and obesity are closely associated with impairments in executive functions among children and adolescents (Liang et al., 2014; Yang et al., 2018; Mamrot and Hanć, 2019).

Executive function, also known as cognitive control or inhibitory control, refers to a set of top-down psychological processes necessary for focusing attention and maintaining concentration (Diamond, 2013). It has a multidimensional structure, with inhibitory control as a key component (Diamond, 2020; Cristofori et al., 2019). Inhibitory control refers to the ability to suppress impulsive responses, automatic behaviors, or distractions from irrelevant stimuli (Diamond, 2013). In the absence of inhibitory control, individuals are driven by impulses, ingrained thought patterns, habitual behaviors (conditioned responses), and external stimuli that continuously capture their attention (Diamond, 2013). Studies have shown that overweight and obese children and adolescents exhibit significant impairments in inhibitory control compared to their normal-weight peers (Yang et al., 2018). Longitudinal studies have also identified a correlation between poor inhibitory control in early childhood and higher BMI in later years (Guxens et al., 2009). This suggests that inhibitory control plays a critical role in weight regulation among children and adolescents (Pauli-Pott et al., 2010). Children with poor inhibitory control are more likely to engage in behaviors associated with obesity, such as overeating, preferring high-fat and high-sugar foods, underconsuming vegetables, and leading a sedentary lifestyle (Pieper and Laugero, 2013; Fogel et al., 2019; Wirt et al., 2015). Therefore, improving inhibitory control in overweight and obese children and adolescents may be a key strategy for promoting healthier lifestyles and preventing or intervening in obesity.

Exercise interventions, which are safe, low-cost, and widely promotable, are considered an ideal approach to improving inhibitory control (Hillman et al., 2008). Neuroimaging and behavioral research demonstrate that regular exercise positively impacts cognitive and executive functions (including inhibitory control) by enhancing prefrontal cortex activity, promoting the release of brain-derived neurotrophic factor (BDNF), improving cerebral blood flow, and fostering neural plasticity (Voss et al., 2011; Dishman et al., 2006; Gomez-Pinilla and Hillman, 2013; Chaddock-Heyman et al., 2013). Several meta-analyses have shown that exercise interventions significantly improve inhibitory control in children and adolescents (Amatriain-Fernández et al., 2021; Song et al., 2025). However, research specifically targeting overweight and obese children and adolescents is limited, with only one available study (Chen et al., 2025). Although this study confirmed a moderate positive effect of exercise on inhibitory control in overweight and obese children and adolescents (SMD = −0.65), there are several important limitations. First, the study treated multiple intervention groups from the same original studies as independent, which violated the assumption of statistical independence in traditional meta-analysis, where each study should ideally contribute only one independent effect size (Chandler et al., 2019; Borenstein et al., 2021). This practice may lead to an underestimation of standard errors and an overestimation of effect sizes and statistical significance (Harrer et al., 2021). Second, the study combined both acute and chronic exercise interventions, which are distinct intervention types. Merging them for effect size estimation could introduce additional heterogeneity and increase the uncertainty in interpreting the results of the moderation analysis. Most importantly, the study did not systematically address the critical issue of “optimal exercise dosage.”

In conclusion, the effects of exercise interventions on inhibitory control in overweight and obese children and adolescents remain unclear, and there is a lack of systematic exploration of exercise dosage and other key moderating variables. Therefore, high-quality meta-analyses are urgently needed to fill this gap, provide evidence-based recommendations for clinical practice, and support the development of more precise and effective intervention programs. This study uses a three-level meta-analysis to integrate potential moderating factors, quantify the effects of exercise interventions on inhibitory control in overweight and obese children and adolescents, and aims to more accurately estimate the true effect of exercise interventions and identify the “optimal dosage” that is most beneficial for improving inhibitory control. The findings are expected to provide valuable evidence for clinical practice and school-based sports interventions and offer scientific guidance for developing more precise and actionable exercise prescriptions.

## 2 Methods

### 2.1 Protocol and registration

This review adhered to the Preferred Reporting Items for Systematic Reviews and Meta-Analyses (PRISMA) guidelines (Chandler et al., 2019). The study protocol was registered in the PROSPERO database prior to initiation, with submission completed by October 2025 (registration number: CRD420251163768).

### 2.2 Information sources and search strategies

Two researchers (PW and DL) independently conducted the literature screening, with the process and final inclusion results cross-checked and confirmed. The literature search was conducted up to October 2025, covering the following databases: PubMed, Web of Science, Embase, Cochrane Library, and CNKI. Detailed Boolean search strategies for each database are provided in Supplementary materials 1.1, with the specific search results presented in Supplementary materials 1.2.

### 2.3 Selection criteria

The literature was systematically screened and evaluated based on the PICOS framework to determine its adherence to predefined inclusion and exclusion criteria (outlined in Supplementary materials 1.3).

1. Participants: Studies included children and adolescents aged 6–18 years, diagnosed with overweight or obesity.
2. Intervention: The studies must have included at least one exercise intervention group, with various exercise types permitted. Studies combining exercise with non-exercise interventions were excluded. Studies focused on acute exercise effects were also excluded.
3. Control Group: Studies must include a control group that did not receive exercise interventions.
4. Outcome: The primary outcome measure was inhibitory control.
5. Study Design: Only experimental studies employing pre- and post-test designs were included, such as randomized controlled trials (RCTs) and quasi-experimental studies.
6. Studies must be published in English or Chinese.
7. Studies must be published in peer-reviewed academic journals.
8. Studies must provide sufficient information for calculating effect sizes.
9. Studies must involve human participants.

### 2.4 Study selection and data processing

The literature search and screening were carried out in multiple stages. Prior to formal screening, one researcher (PW) was responsible for literature retrieval and initial deduplication. Deduplication was first performed using the automatic deduplication feature of EndNote X9 (Hupe, 2019). Subsequently, any remaining duplicates not identified by the software were manually checked and removed. The remaining studies were then exported and imported into the Rayyan platform for further screening (Ouzzani et al., 2016). Two researchers (PW and DL) independently reviewed the titles and abstracts according to the predefined inclusion and exclusion criteria. For disagreements of less than 1%, consensus was reached through discussion. Studies that met the inclusion criteria were advanced to the full-text assessment stage, which was independently conducted by two researchers (PW and DL). In cases of disagreement, a third researcher (JH) participated in the discussion until a consensus was reached.

Data extraction was independently conducted by two researchers (PW and DL) using a pre-designed, standardized data extraction form. The consistency rate for data extraction was 95.08%. For any discrepancies, discussions were held between the two researchers, and if consensus could not be reached, a third researcher (JH) was involved in the discussion to resolve the issue. The extracted data included basic study information (authors and publication year) and moderating variables. These moderating variables were categorized according to the PICO framework (Akobeng, 2005), which is commonly used in evidence-based medicine, into population, intervention, control, and outcome variables. Specific extraction criteria for the moderating variables are presented in Table 1, with further details available in Supplementary materials 1.4.

**Table 1:** Standardized protocol used for data extraction from the selected studies.

PICO	Moderator	Category
Population	Age	Children (6–11 years old); Adolescents (12–18 years old).
Intervention	Type of motor skills	Open motor skills; Closed motor skills
Intervention	Training frequency	Two times per week; Three times per week; Five times per week; Ten times per week.
Intervention	Training intensity	MPA; MVPA; VPA.
Control	Control group	Active control group (receiving additional non-exercise intervention programs); Passive control group (no additional intervention, only participating in pre-test and post-test).
Outcome	Task performance metrics	Time; Accuracy; Score.

### 2.5 Statistical analysis

Because a single study often includes multiple outcome measures, which may lead to dependencies between effect sizes and violate the assumption of independence in meta-analysis, this study employed a three-level meta-analysis model (Cheung, 2019). This approach effectively addresses the dependency issue and allows for the inclusion of more effect sizes (Cheung, 2019). Following established guidelines and prior literature, Hedges’ g was chosen as the statistical measure for intervention effects (Assink and Wibbelink, 2016; Cheung, 2014; Cui et al., 2024; Borenstein et al., 2010), where positive values indicate better improvement in inhibitory control for the experimental group compared to the control group. During the data preprocessing phase, effect sizes and their variances were calculated using the method proposed by Aksayli et al. in Excel (Aksayli et al., 2019). The core statistical analysis was then performed using the metafor package in R (R Core Team, 2024). For the specific calculation formulas and detailed statistical process, refer to Supplementary materials 1.5.

### 2.6 Risk of bias assessment

The risk of bias for the included studies was assessed using the Cochrane Risk of Bias 2.0 (RoB2) tool, evaluating five core dimensions of bias risk: bias due to the randomization process, bias from deviations from intended interventions, bias due to missing outcome data, bias in the measurement of outcomes, and bias from selective reporting of results (Sterne et al., 2019). Based on this assessment, each study was classified as having “low risk of bias,” “some concerns,” or “high risk of bias (Sterne et al., 2019).” This evaluation was independently carried out by two researchers (PW and DL), and in cases of disagreement, a third researcher (JH) was involved to reach a consensus.

### 2.7 Quality of evidence

The overall quality of evidence was assessed using the GRADE framework, with GRADEpro software (Balshem et al., 2011). The evaluation considered five factors: study design limitations (i.e., risk of bias), inconsistency, indirectness, imprecision, and publication bias (Guyatt et al., 2011). Each dimension was rated as “not serious” (no downgrade), “serious” (one level downgrade), or “very serious” (two levels downgrade) (Guyatt et al., 2011). Based on this evaluation, the overall quality of evidence for each outcome was classified as high, moderate, low, or very low (Guyatt et al., 2011). It is important to note that if fewer than 10 studies were included, the “publication bias” dimension was excluded from the GRADE assessment (Afonso et al., 2024). Consequently, publication bias was not considered in the evaluation of the quality of evidence for the moderating variables in this study.

## 3 Results

**Figure 1:**
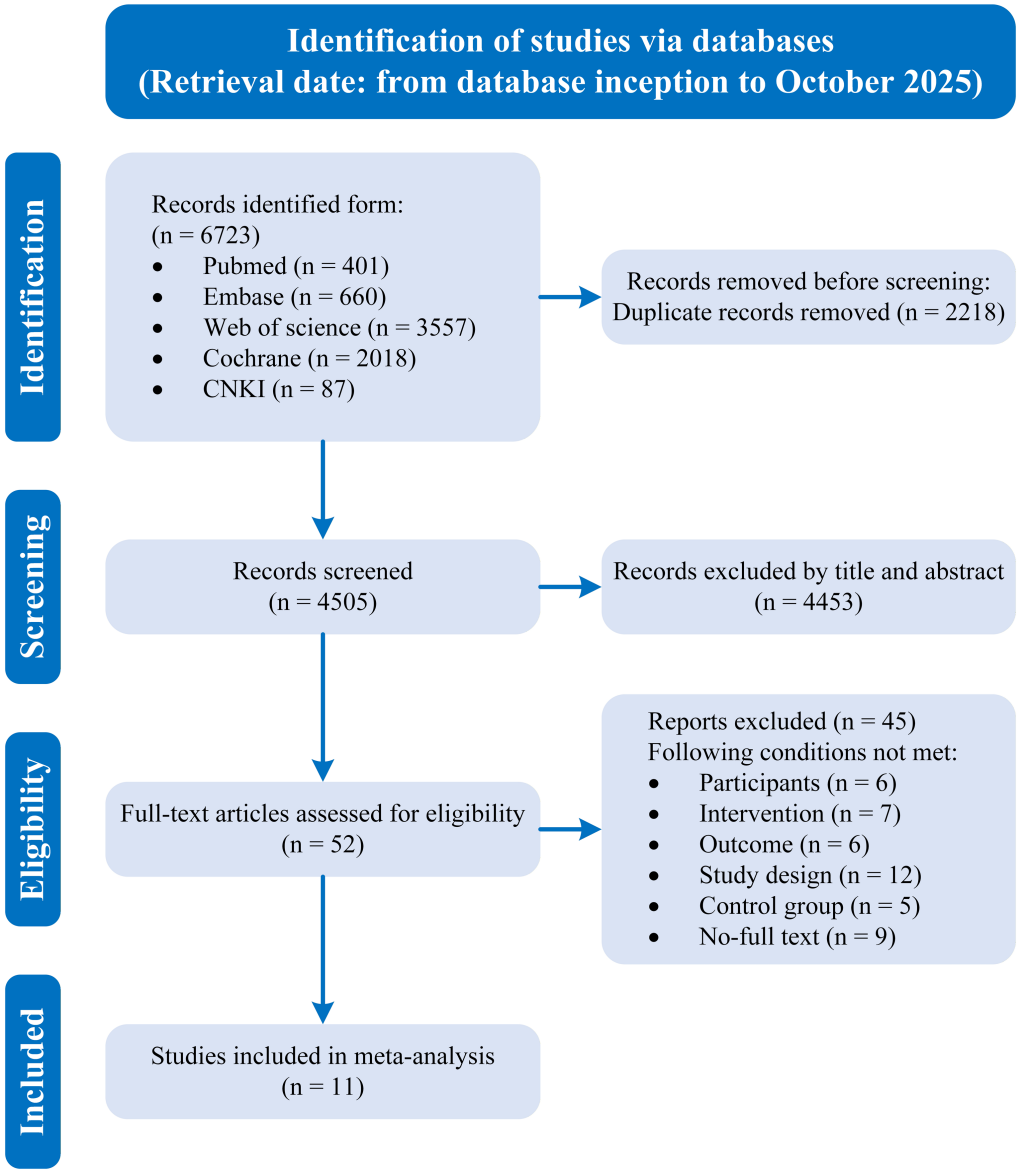
PRISMA flow diagram of the study process.

As shown in Figure 1, a total of 6,723 articles were retrieved from five databases. Of these, 2,218 were removed due to duplication. After screening the titles and abstracts, 52 articles advanced to full-text evaluation, and ultimately, 11 studies met the inclusion criteria and were included for data extraction and analysis. The total sample size across the included studies was 787 children and adolescents, divided into an experimental group (448 participants, 57%) and a control group (339 participants, 43%). The participants’ ages ranged from 8.75 (Logan et al., 2021) to 14.06 (Liu et al., 2018) years. The studies were published between 2014 and 2025. Geographically, the studies originated from various countries and regions: 6 from China (Liu et al., 2018; Chou et al., 2023; Chou et al., 2019; Wang et al., 2024; Yuan et al., 2025; Shang et al., 2020), 2 from the United States (Logan et al., 2021; Krafft et al., 2014), 2 from Spain (Mora-Gonzalez et al., 2024; Ortega et al., 2022), and 1 from Thailand (Intawachirarat et al., 2025). Detailed characteristics of the studies are provided in Supplementary materials 2.1.

### 3.1 The overall effect of exercise intervention on inhibitory control

To evaluate the overall effect of exercise interventions on inhibitory control, we combined all effect sizes. The three-level random-effects model showed a significant overall effect (*g* = 0.45, 95% Confidence Interval (CI) = [0.14, 0.76], 95% Prediction Interval (PI) = [−0.72, 1.62], *p* = 0.005). Similarly, the two-level random-effects model also showed a significant overall effect (*g* = 0.55, 95% CI = [0.35, 0.74], 95% PI = [−0.76, 1.86], *p* < 0.001). Cochran’s Q statistic indicated significant heterogeneity (*Q*(52) = 278.81, *p* < 0.001).

### 3.2 Influence analysis

**Figure 2:**
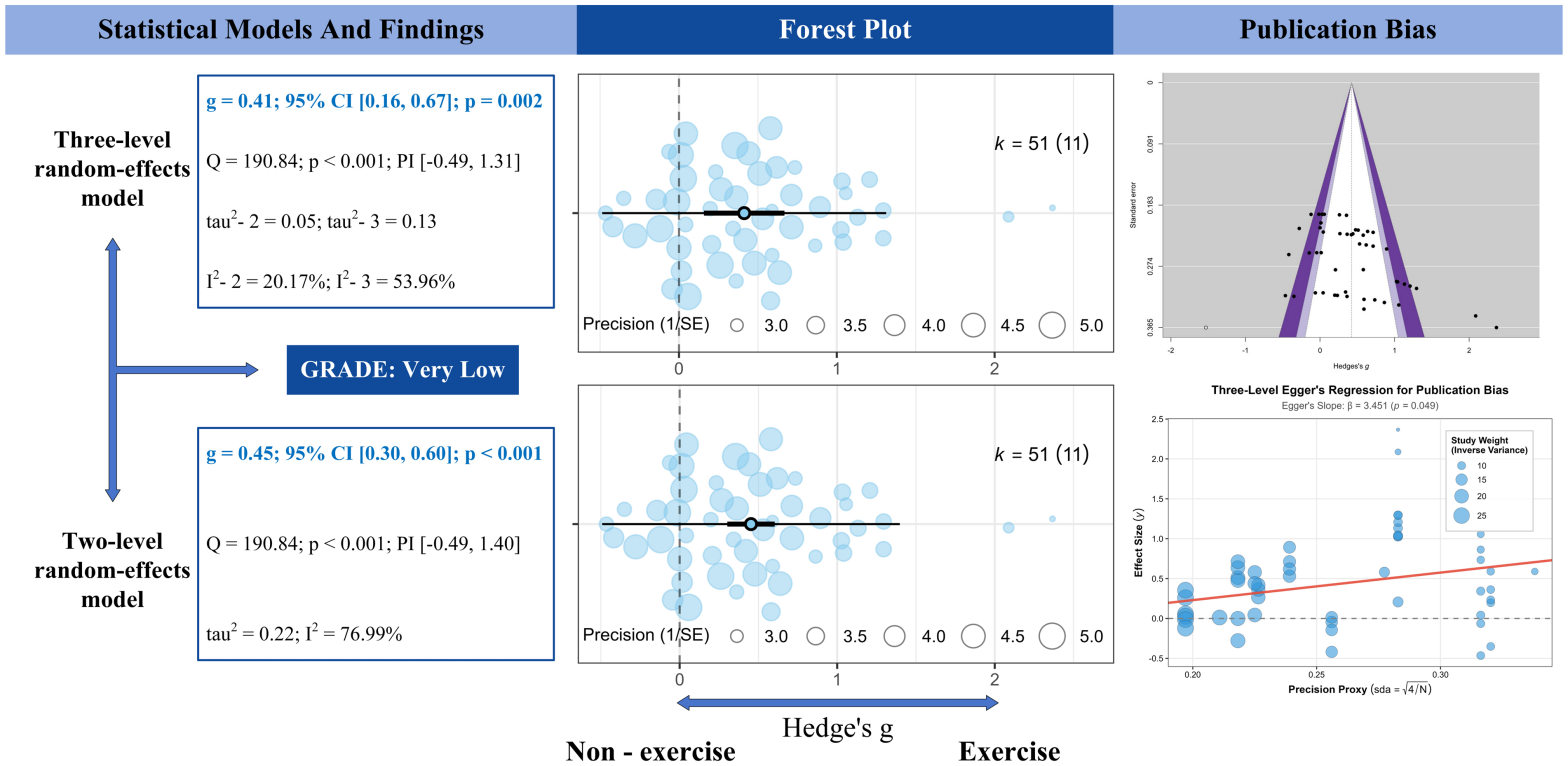
Overall effect of exercise intervention on inhibitory control. CI, confidence interval; PI, prediction interval; k represents the number of effect sizes (number of studies).

To test for potential outliers that could distort the results of the meta-analysis, we conducted an influence analysis and identified two outliers (16: *g* = 3.04, 22: *g* = 3.45 (Chou et al., 2023); see Supplementary Figure S5) (Viechtbauer and Cheung, 2010). After excluding these outliers, we performed another round of meta-analysis (see Figure 2). The results showed that although the overall effect size in the three-level random-effects model slightly decreased, it remained significant (*g* = 0.41, 95% CI = [0.16, 0.67], 95% PI = [−0.49, 1.31], *p* = 0.002). The two-level random-effects model yielded similar results, with a slight decrease in the overall effect size but still significant (*g* = 0.45, 95% CI = [0.30, 0.60], 95% PI = [−0.49, 1.40], *p* < 0.001). Cochran’s Q statistic, though slightly reduced, still indicated significant heterogeneity (*Q*(50) = 190.84, *p* < 0.001). To mitigate the influence of outliers, we excluded them in subsequent studies.

### 3.3 Heterogeneity analysis

After removing outliers, heterogeneity remained significant, with *Q*(50) = 190.84, *p* < 0.001. In the total variation, the sampling variance (level 1) accounted for 25.87%, within-study variance (level 2) accounted for 20.17%, and between-study variance (level 3) accounted for 53.96%. The likelihood ratio test (LRT) showed that the within-study variance (level 2) was significant (LRT = 6.68, *p* = 0.01, two-tailed), indicating that the three-level model was superior to the two-level model with level 2 set to 0. Similarly, the between-study variance (level 3) was significant (LRT = 25.38, *p* < 0.001, two-tailed), showing that the three-level model outperformed the two-level model with level 3 set to 0. These results support the necessity of using a three-level model. Detailed results can be found in Supplementary Tables S8 and S9. In meta-analysis, when multiple effect sizes are reported within a study, a nested structure exists between the effect sizes and studies (Assink and Wibbelink, 2016). The three-level model more accurately captures the hierarchical structure of the data, fully accounting for the nested relationships between effect sizes within studies and between studies (Harrer et al., 2021). Based on this, subsequent analyses in this study employed the three-level random-effects model.

### 3.4 Moderator analysis

**Figure 3:**
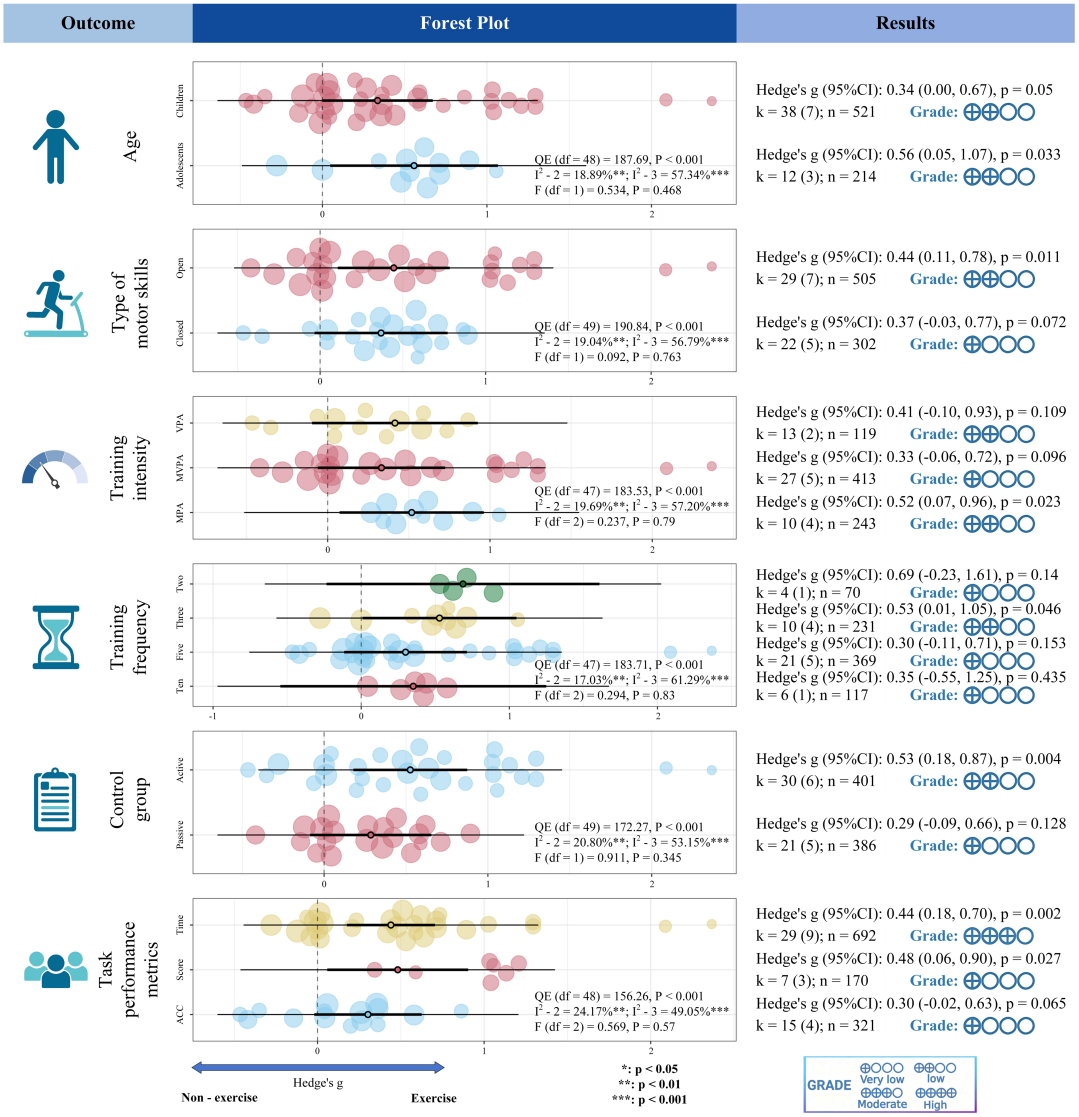
The results of moderators of the effect of exercise intervention on inhibitory control. CI, confidence interval; k represents the number of effect sizes (number of studies); n represents the sample size. Created in BioRender. Dong (2026) https://BioRender.com/5odtr0f.

This study systematically evaluated the effects of a series of potential moderating variables on the overall effect size, including age, type of motor skills, training intensity, training frequency, control group, and task performance metrics (see Figure 3). However, statistical analyses showed that none of the pre-specified moderating variables reached statistical significance. In addition, the Benjamini–Hochberg false discovery rate (FDR) correction method was further applied to the omnibus test *p*-values of all moderator analyses to account for multiple comparisons. After correction, the results remained unchanged, and none of the moderating variables reached statistical significance (*p* > 0.05; see Supplementary Table S10). The absence of significant moderating effects may be related to insufficient statistical power resulting from the relatively limited number of studies included in the present meta-analysis, rather than the true absence of moderating effects. Therefore, future studies with larger sample sizes and higher methodological quality are still needed to further verify the potential moderating roles of these variables.

### 3.5 Meta-regression

To explore the potential “optimal dosage effect,” a three-level meta-regression analysis using restricted cubic splines (RCS) was conducted to clarify the nonlinear relationship between exercise dosage and effect size, and to identify potential dosage thresholds. To determine the optimal number of knots for the RCS model, models with 3, 4, and 5 knots were compared. Because models with different knot numbers contain different fixed-effect structures, maximum likelihood estimation (ML) was used for model fitting, and model fit was evaluated using likelihood ratio tests (LRT), Akaike information criterion (AIC), and Bayesian information criterion (BIC).

For training sessions, the 4-knot model demonstrated a significantly better fit than the 3-knot model (LRT = 13.53, *p* < 0.001), whereas increasing the model complexity to 5 knots did not further improve model fit (LRT < 0.001, *p* > 0.99). Moreover, the 4-knot model showed the lowest AIC (68.93) and BIC (80.52) values. Therefore, the 4-knot model was selected for subsequent dose–response analyses of training sessions. For training duration, the 4-knot model also significantly improved model fit compared with the 3-knot model (LRT = 13.22, *p* < 0.001). In addition, the 5-knot model further improved model fit relative to the 4-knot model (LRT = 10.75, *p* = 0.001), while also yielding the lowest AIC (54.91) and BIC (68.43) values and reducing residual heterogeneity (QE = 89.27). Accordingly, the 5-knot model was selected to characterize the dose–response relationship for training duration. Detailed model comparison results are presented in Supplementary Tables S11 and S12.

**Figure 4:**
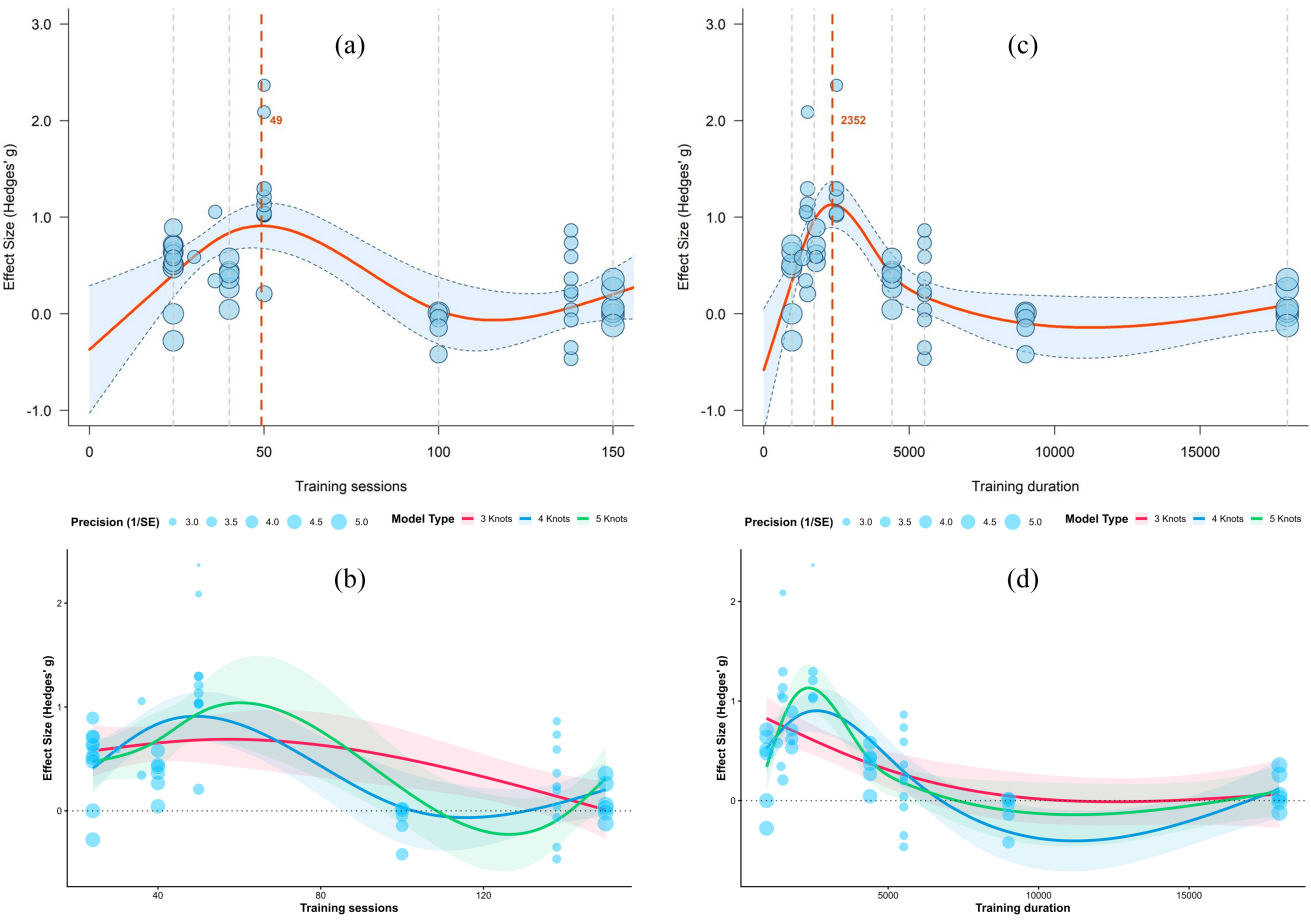
Nonlinear dose–response relationships between exercise intervention and inhibitory control. **(a)** Effect of training sessions on inhibitory control: this panel illustrates the nonlinear relationship between the number of training sessions and inhibitory control, with the optimal number of sessions identified at 49. The dashed vertical lines represent knot locations at 24, 40, 100, and 150 sessions; **(b)** Effect of training sessions across different models: this panel compares the effect of training sessions on inhibitory control using models with 3, 4, and 5 knots. The colored lines represent the different models, and the shaded areas indicate the 95% confidence intervals; **(c)** Effect of training duration on inhibitory control: this panel shows the relationship between total training duration and inhibitory control, with the optimal duration identified at 2,352 min. Knot locations are marked at 960, 1725, 4,400, 5,520,and 18,000 min; **(d)** Effect of training duration across different models: similar to panel (b), this panel compares the effect of training duration on inhibitory control across models with 3, 4, and 5 knots, illustrating how different model choices influence the estimated effect size.

For training sessions, the spline term moderation factor test showed statistical significance for all spline coefficients [*F*(3, 47) = 10.43, *p* < 0.001], indicating that the number of training sessions significantly influenced the intervention effects. However, the residual heterogeneity Q statistic (QE) remained significant [QE(df = 47) = 122.42, *p* < 0.001]. The fitted curve suggested that exercise interventions appeared to reach peak effectiveness after approximately 49 training sessions in overweight and obese children and adolescents (see Figures 4A,B). Similarly, the moderation factor test for training duration was also statistically significant [*F*(4, 46) = 14.76, *p* < 0.001], although the residual heterogeneity Q statistic remained significant [QE(df = 46) = 89.27, *p* = 0.001]. The fitted curve suggested that peak effectiveness was achieved when the total training duration reached approximately 2,352 min (see Figures 4C,D).

To further examine the robustness of the dose–response relationship, sensitivity analyses were conducted by excluding studies with extreme intervention doses (i.e., 150 training sessions and 18,000 total training minutes). After excluding these studies, the nonlinear associations for both training sessions [*F*(3, 41) = 4.33, *p* = 0.01] and training duration [*F*(4, 40) = 10.19, *p* < 0.001] remained statistically significant. The estimated optimal dosage changed slightly, with peak effectiveness observed at approximately 41 training sessions and 2,285 total training minutes (see Supplementary Tables S8, S9). Although the estimated optimal values shifted modestly after excluding extreme-dose studies, the overall dose–response pattern remained largely consistent, suggesting that the findings were relatively robust and not solely driven by extreme intervention doses.

In summary, based on the current dose–response analyses and available evidence, the optimal intervention dosage for improving inhibitory control in overweight and obese children and adolescents appears to be approximately 49 training sessions, with each session lasting about 48 min. However, these findings should be interpreted cautiously rather than as definitive exercise prescriptions, and future large-scale, high-quality randomized controlled trials are still needed to further validate and refine these preliminary dosage recommendations.

**Figure 5:**
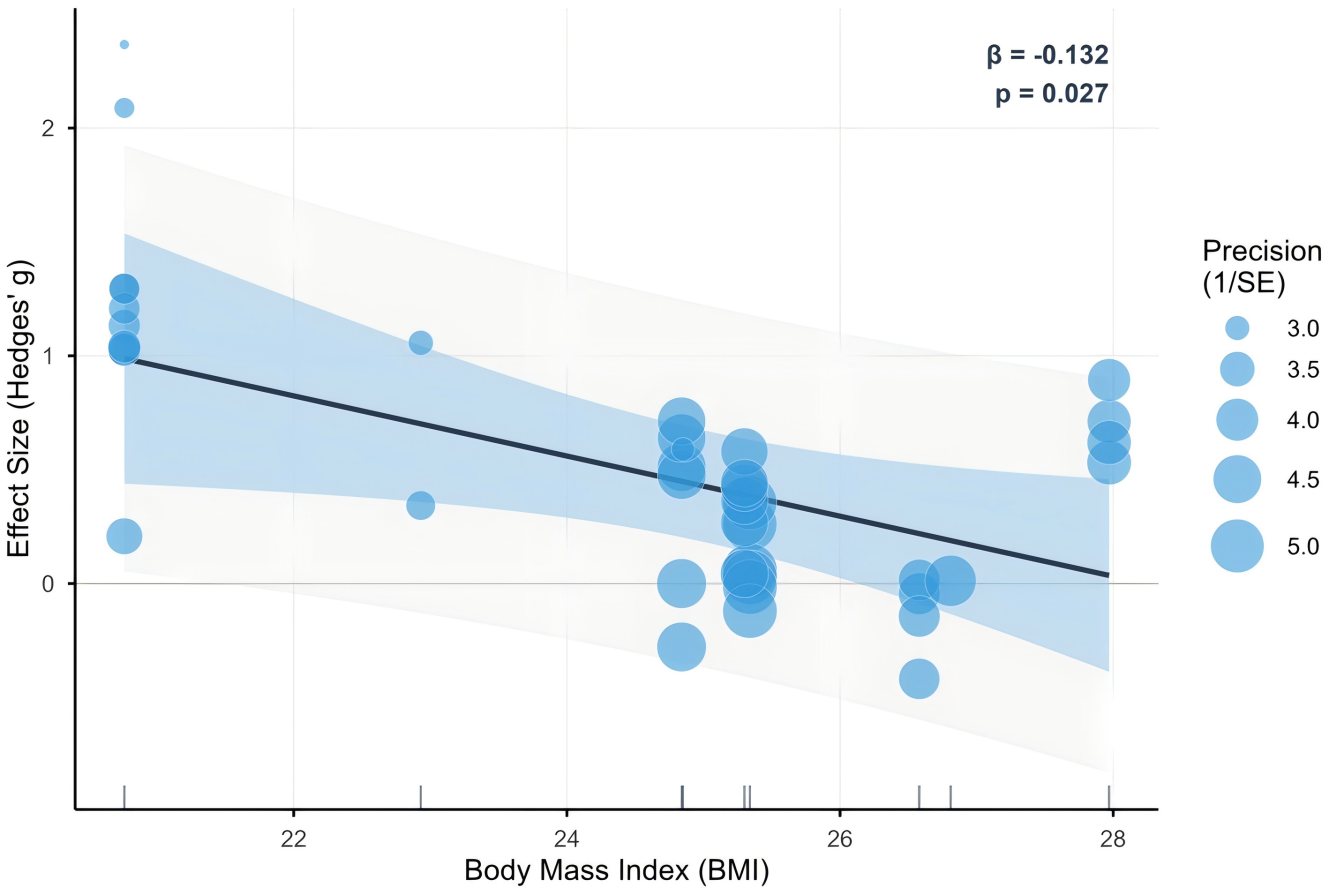
Meta-regression analysis of the effect of baseline BMI on intervention outcomes.

To investigate the effect of participants’ baseline BMI on the intervention effect, a three-level meta-regression model was constructed. The results showed a statistically significant linear association between BMI and effect size (*F*(1, 38) = 5.31, *p* = 0.027). The regression coefficient was *β* = −0.13 (95% CI: −0.25, −0.02), indicating that a higher baseline BMI was associated with a smaller intervention effect (a significant negative correlation). Although BMI explained part of the variation, the residual heterogeneity test remained significant (QE(38) = 115.12, *p* < 0.001) (see Figure 5).

### 3.6 Risk of bias

We assessed the quality of the included studies using the RoB 2 tool. Among the 11 studies, 2 (18.18%) were rated as high risk, while 9 (81.81%) were rated as having “some concerns” (see Supplementary Figures S6, S7). It is important to note that the majority of studies were rated as having “some concerns” for two primary reasons. First, most studies only mentioned “random allocation” in their description of the randomization process, but did not report the specific method used for random sequence generation and rarely mentioned allocation concealment. This raised concerns about “bias in the randomization process.” Second, many studies lacked pre-registration, and the assessment of “bias from selective reporting of results” relies on pre-registered protocols to verify pre-specified outcomes and analyses. Consequently, this item was also rated as “some concerns.” Two studies were rated as high risk. One study had missing results (Liu et al., 2018), which could potentially affect the true values, while the other study deviated from the expected intervention due to the experimental environment (Mora-Gonzalez et al., 2024), with these biases not being evenly distributed across the groups.

### 3.7 Publication bias

We assessed publication bias using funnel plots (Sterne and Egger, 2001), Egger’s regression test (Egger et al., 1997), and the trim-and-fill method (Duval and Tweedie, 2000). First, the funnel plot for the two-level model showed asymmetry, suggesting a potential risk of publication bias (see Figure 2). Egger’s regression test results were significant for the two-level model (*t*(49) = 3.86, *p* < 0.001); in the multi-level Egger test with modified prediction factors, the results were also significant (*t*(49) = 2.02, *p* = 0.049). The trim-and-fill method using the “R0” method showed that one effect size might be missing on the left side of the mean effect size. After correction, the overall effect size remained statistically significant (*g* = 0.42, 95% CI [0.27, 0.58], *p* < 0.001). In conclusion, although there may be a high risk of publication bias in this analysis, it did not have a significant impact on the results.

### 3.8 Level of evidence quality assessment

The certainty of evidence was assessed using the GRADE framework (Guyatt et al., 2011). Due to limitations in the design of the included original studies (risk of bias), significant heterogeneity between studies (high heterogeneity), and potential publication bias, the level of evidence was downgraded. The final evaluation showed that the quality of evidence supporting the conclusion that exercise interventions improve inhibitory control was classified as “Very Low.” The evaluation of moderating variables is presented in Supplementary Table S13.

## 4 Discussion

This study employed a three-level meta-analysis to integrate data from 11 experimental studies (N = 787), systematically assessing the impact of exercise interventions on inhibitory control in overweight and obese children and adolescents. Furthermore, the study explored the “optimal intervention dosage” of exercise interventions using the restricted cubic spline (RCS) model. The meta-analysis revealed that exercise interventions significantly improved inhibitory control in this population. Notably, this is the first study to identify a nonlinear trend in exercise intervention effects based on dose–response analysis and to highlight baseline BMI as a significant moderating factor influencing intervention outcomes through meta-regression. Based on the available evidence, the results suggest that approximately 49 exercise sessions, each lasting 48 min, may represent the optimal dosage for improving inhibitory control in overweight and obese children and adolescents. Moreover, individuals with lower baseline BMI tend to experience more substantial improvements from the intervention.

### 4.1 Effect of exercise interventions on inhibitory control

Although the overall effect size of exercise interventions was moderate (*g* = 0.41) and statistically significant (Cohen, 2013), it is important to note the considerable heterogeneity among studies. From a practical perspective, a Hedges’ g of 0.41 indicates a modest yet potentially meaningful improvement in inhibitory control performance. In practical applications, this magnitude of effect may translate into measurable improvements in classic inhibitory control paradigms, such as reduced reaction time interference during the Stroop color-word task or fewer commission errors during Go/No-Go and Stop-Signal tasks. For overweight and obese children and adolescents, who often exhibit baseline deficits in inhibitory control, such improvements may contribute to better behavioral self-regulation and executive functioning in daily life. However, the practical interpretation of this effect should be balanced against the wide prediction interval, which includes negative values. This finding suggests that the effectiveness of exercise interventions may vary substantially across different intervention settings, participant characteristics, and implementation contexts. In some real-world scenarios or specific subpopulations, the actual benefits of exercise interventions may be limited or potentially absent. Therefore, caution is warranted when generalizing the pooled effect estimate to all populations and intervention conditions. Despite this variability, the conclusion that exercise interventions significantly improve inhibitory control remains robust, as indicated by the average effect from the pooled data. First, after removing outliers, 22 of the 51 effect sizes from the original studies had entirely significant confidence intervals, and four effect sizes had confidence intervals near the significance threshold, suggesting a consistent trend across studies. The overall significant effect was not driven by a few extreme values. Second, the result remained statistically significant in both the three-level and two-level models, and after correcting for publication bias using the trim-and-fill method, the effect size remained robust. Third, compared to the only existing meta-analysis on this topic, this study addressed the issue of dependency between multiple effect sizes in original studies by using a three-level model, which prevents the underestimation of standard errors and the overestimation of statistical significance, thus providing more accurate and conservative estimates of effect sizes (Harrer et al., 2021; Cheung, 2019).

This finding provides further support for the neurobiological hypothesis that exercise can improve cognitive function (Hillman et al., 2008). Overweight and obesity in children and adolescents are closely associated with impairments in executive functions, including inhibitory control. This association may be mediated by structural changes in the prefrontal cortex (PFC), such as reductions in cortical thickness (Ronan et al., 2020). Previous neuroimaging studies have shown that overweight and obese children and adolescents often exhibit structural or functional alterations in the PFC, particularly widespread reductions in cortical thickness (Ronan et al., 2020; Laurent et al., 2020). As the PFC is crucial for inhibitory control, improving PFC-related neural functions through effective interventions may enhance inhibitory control in this population.

Several mechanisms may underlie the observed improvements in inhibitory control. At the neurobiological level, regular exercise has been shown to stimulate the release of brain-derived neurotrophic factor (BDNF) and insulin-like growth factor-1 (IGF-1), which promote neurogenesis, synaptic formation, and neural plasticity (Cotman and Berchtold, 2002; Hötting and Röder, 2013). These processes provide a biological foundation for the structural and functional recovery of executive function networks, including the PFC. Exercise also increases cerebral blood flow and improves the metabolic efficiency of oxygen and glucose (Kleinloog et al., 2019; Kleinloog et al., 2022), which may enhance the functional activation of the PFC during executive function tasks (Endo et al., 2013).

From a psychological perspective, overweight and obese children and adolescents often experience higher levels of psychological stress and are more prone to negative emotional states such as anxiety and sadness (Dong et al., 2023; Moradi et al., 2021). When individuals are under prolonged stress or negative emotions, the PFC and related executive functions are typically the first to be affected (Diamond and Ling, 2016). Conversely, when stress levels are lower, and emotional and physical health are improved, executive functions, including inhibitory control, tend to perform better (Diamond and Ling, 2016). In this context, exercise interventions may help alleviate psychological stress and improve emotional states, which can, in turn, restore PFC-related inhibitory control (Chen et al., 2024; Cai et al., 2025).

Additionally, exercise may indirectly improve inhibitory control by reducing chronic low-grade inflammation associated with obesity. Overweight and obese children and adolescents often have elevated levels of inflammatory markers, which negatively affect the structure and function of the PFC (Miller and Spencer, 2014; Guillemot-Legris and Muccioli, 2017), thereby impairing inhibitory control. Regular exercise has been shown to reduce inflammation and improve neuroinflammatory status (Gleeson et al., 2011), creating a more favorable physiological environment for the normal functioning of the PFC and executive functions.

### 4.2 Dose–response relationship and moderator effect analysis

To address clinical uncertainties regarding the development of “exercise prescriptions,” this study employed the restricted cubic spline (RCS) model to explore the nonlinear relationship between intervention dosage and effect size. The results revealed a clear threshold effect and plateau phase in the improvement of inhibitory control due to exercise interventions.

Specifically, the study found that the “optimal dosage” for improving inhibitory control in overweight and obese children and adolescents was approximately 49 training sessions, each lasting about 48 min. This suggests that the relationship between intervention dosage and effect size is not a simple linear positive correlation (Tsukamoto et al., 2017). However, sufficient training accumulation is necessary to induce stable structural and functional adaptations in the brain (Krafft et al., 2014; Davis et al., 2011; Krafft et al., 2014). Short-term or sporadic exercise may also produce some immediate intervention effects, but these improvements are likely to be short-lived, typically lasting no more than 1–2 h (Diamond and Ling, 2016), and insufficient to trigger long-term neural remodeling (Basso et al., 2015). It is important to note that the dose–response curve does not follow a “more is better” linear pattern. This phenomenon may be attributed to physical fatigue or psychological burnout induced by excessive training. Overly intense or excessive training may not only reduce participant adherence but could also lead to negative emotional responses, which may counteract the benefits of exercise on inhibitory control (Meeusen et al., 2013; Singh et al., 2025). Furthermore, during the early stages of intervention, participants must learn and master new techniques or rules, which often requires higher cognitive engagement (Dayan and Cohen, 2011; Floyer-Lea and Matthews, 2005). As training progresses, motor skills become more automated, and the activation of brain regions involved in inhibitory control, such as the prefrontal cortex, decreases (Sakai et al., 1998; Tomporowski and Pesce, 2019). This may lead to a plateau in intervention effects. These findings align with Diamond’s theoretical framework (Diamond and Ling, 2016), which posits that improvements in executive function depend on sustained and challenging task stimuli. Once the training duration reaches a certain threshold, without an increase in task difficulty or cognitive challenge, further improvements in executive function are unlikely. Nevertheless, the identified optimal dosage should be interpreted cautiously. The estimated peak effect was located relatively close to the upper boundary of the currently available data range, and the number of studies involving higher-dose interventions remained limited. Therefore, it remains unclear whether the observed plateau reflects a true neuroadaptive ceiling effect or is partially attributable to sparse data at higher intervention doses. Although the sensitivity analyses excluding extreme-dose studies yielded broadly consistent findings, future large-scale randomized controlled trials examining higher-dose exercise regimens are still needed to further validate the stability of the dose–response relationship and refine the current dosage recommendations.

The meta-regression analysis also revealed that baseline BMI is a significant moderating factor for improvements in inhibitory control through exercise interventions, with a negative correlation (*β* = −0.13). Specifically, children and adolescents with higher baseline BMI showed smaller improvements in inhibitory control from the exercise intervention. This result may be related to structural brain differences associated with varying weight statuses. Previous studies have indicated that obese adolescents tend to exhibit reduced gray matter volume in certain brain regions, particularly the superior and middle frontal gyri of the prefrontal cortex, which are crucial for inhibitory control (Yokum et al., 2012). Moreover, reduced gray matter volume in brain regions related to inhibitory control has been linked to a higher risk of future weight gain (Yokum et al., 2012). Consistent with these findings, Huang et al. found that children with larger waist circumferences performed worse on inhibitory control tasks (Huang et al., 2015). In older populations, Raji et al. (2010) demonstrated that compared to individuals of normal weight, obese individuals displayed more widespread brain atrophy, affecting areas such as the prefrontal cortex, anterior cingulate gyrus, hippocampus, and thalamus, while overweight individuals predominantly exhibited atrophy in the basal ganglia and white matter regions. Taken together, these findings suggest that higher baseline BMI may be associated with more significant structural changes in the prefrontal cortex, leading to greater impairments in inhibitory control function and reduced neural plasticity. This could, in turn, diminish the brain’s ability to respond to exercise interventions, thereby weakening their effects.

Given that higher baseline BMI is associated with diminished intervention responses, a “one-size-fits-all” approach may be inadequate. We propose the adoption of BMI-stratified exercise prescriptions. Individuals with higher baseline BMI likely require adapted strategies—such as increased training intensity, greater cognitive engagement, and progressive physical–cognitive overload—to achieve neural adaptations comparable to those with lower baseline BMI. This tailored approach may help maintain an optimal challenge range, potentially mitigating the baseline inhibitory control and neural deficits often observed in individuals with obesity.

Although other pre-specified moderating variables did not reach statistical significance, these findings should be interpreted with caution and not taken as evidence of absent moderating effects. The null results may reflect limited statistical power due to the small number of included studies. Accordingly, these analyses should be considered exploratory, and further research based on a larger evidence base is required.

### 4.3 Study limitations and implications

To our knowledge, this is the first study to apply a three-level meta-analysis combined with the restricted cubic spline (RCS) model to systematically explore the dose–response relationship between exercise interventions and inhibitory control in overweight and obese children and adolescents. However, several limitations must be considered. First, the number of studies and sample sizes included were relatively small, and publication bias was present. Although this bias did not significantly affect the results, it may have reduced statistical power. Second, there was considerable heterogeneity among the included studies. While moderator analysis and meta-regression accounted for some of the sources of variation in effect sizes, significant residual heterogeneity remained, suggesting that other unidentified factors may contribute to the observed variation. Third, most of the included studies lacked long-term follow-up data, leaving uncertainty about how long the improvements in inhibitory control persist after the intervention. Fourth, and most importantly, despite our methodological enhancements, the overall certainty of the synthesized evidence evaluated using the GRADE framework remained “very low,” suggesting that the present findings should be interpreted with appropriate caution. Therefore, the suggested optimal dosage of 49 sessions lasting 48 min each should not be considered a definitive exercise prescription. Until further validated by large-scale, high-quality, multi-center randomized controlled trials, these findings should be regarded as hypothesis-generating rather than practice-defining. Given the complex relationship between inhibitory control and weight status, particularly the more severe deficits observed in the obese population, future studies should focus on developing exercise prescriptions tailored to weight stratification, such as different BMI categories. Furthermore, exercise program designs should emphasize a progressive approach to both physical and cognitive load, dynamically increasing task difficulty to keep participants in the “challenge zone.” This strategy could break through the plateau phase of cognitive benefits, thereby maximizing the effectiveness of the intervention.

## 5 Conclusion

Exercise interventions can significantly improve inhibitory control in overweight and obese children and adolescents. Based on the current evidence, this study recommends that exercise interventions be implemented as early as possible when abnormal weight status is identified in children and adolescents. The dose–response analysis suggested that approximately 49 training sessions, each lasting about 48 min, may represent a potentially beneficial intervention dosage for improving inhibitory control. However, given the limited number of available studies and the overall “very low” certainty of evidence according to the GRADE framework, these findings should be interpreted cautiously and regarded as hypothesis-generating rather than definitive clinical recommendations. Therefore, future well-designed, adequately powered randomized controlled trials are needed to further validate and expand upon these findings.
