# Supplementary material for: Optimal dosage of exercise interventions for enhancing inhibitory control in overweight and obese children and adolescents: insights from a multilevel meta-analysis
Source: Front Psychol. 2026 Jun 17;17:1870462. doi: 10.3389/fpsyg.2026.1870462 (PMC13318666; doi:10.3389/fpsyg.2026.1870462)
Supplement: Supplementary file 1 [file Table_1.DOCX]

**Supplementary material** for

**Optimal dosage of exercise interventions for enhancing inhibitory control in overweight and obese children and adolescents: insights from a multilevel meta-analysis**

**1 Supplementary Method**

### 1.1 Characteristics of the studies

**Table S1** Complete search strategy for each database.

| 1. Exercise | Mesh: exercise  TI/AB: exercise* OR sport* OR training* OR aerobic exercis* OR plyometric exercis* OR resistance training* OR strength training* OR weight training* OR physical activit* OR stretch* OR muscle stretch* OR jogging OR walking OR ambulation OR running OR swimming OR dancing OR cycling OR biking OR yoga OR pilates OR tai ji OR taijiquan OR qi gong OR qigong OR baduanjin OR exergam* OR treadmill* OR stair climbing OR endurance training* OR athletic* OR high-intensity interval training* OR HIIT |
| --- | --- |
| 2. Overweight or obesity | Mesh: overweight OR obesity  TI/AB: overweight OR obes* OR excess weight OR excess body weight OR high body mass index OR high BMI |
| 1. Population | Mesh: child OR adolescent  TI/AB: child* OR adolescen* OR youth* OR teen* OR pediatric* OR paediatric* OR school-age* OR young people OR young person* |
| 4. Inhibitory control | Mesh: executive function  TI/AB: inhibitory control OR response inhibition OR behavioral inhibition OR behavioural inhibition OR cognitive inhibition OR interference control OR impulse control OR impulsiv* OR self-control OR self control OR executive function* OR executive control* OR executive functioning OR cognitive function* OR cognitive control* OR cognitive abiliti* OR cognition |
| 1 AND 2 AND 3 AND 4 | |

## 1.2 Search results

**Table S2** Pubmed

| Step | Amount | Search string |
| --- | --- | --- |
| #1 | [281,147](https://pubmed.ncbi.nlm.nih.gov/?term=exercise%5bMeSH+Terms%5d&ac=no&sort=relevance) | exercise[MeSH Terms] |
| #2 | [1,612,652](https://pubmed.ncbi.nlm.nih.gov/?term=exercise*%5bTitle/Abstract%5d+OR+sport*%5bTitle/Abstract%5d+OR+training*%5bTitle/Abstract%5d+OR+aerobic+exercis*%5bTitle/Abstract%5d+OR+plyometric+exercis*%5bTitle/Abstract%5d+OR+resistance+training*%5bTitle/Abstract%5d+OR+strength+training*%5bTitle/Abstract%5d+OR+weight+training*%5bTitle/Abstract%5d+OR+physical+activit*%5bTitle/Abstract%5d+OR+stretch*%5bTitle/Abstract%5d+OR+muscle+stretch*%5bTitle/Abstract%5d+OR+jogging%5bTitle/Abstract%5d+OR+walking%5bTitle/Abstract%5d+OR+ambulation%5bTitle/Abstract%5d+OR+running%5bTitle/Abstract%5d+OR+swimming%5bTitle/Abstract%5d+OR+dancing%5bTitle/Abstract%5d+OR+cycling%5bTitle/Abstract%5d+OR+biking%5bTitle/Abstract%5d+OR+yoga%5bTitle/Abstract%5d+OR+pilates%5bTitle/Abstract%5d+OR+tai+ji%5bTitle/Abstract%5d+OR+taijiquan%5bTitle/Abstract%5d+OR+qi+gong%5bTitle/Abstract%5d+OR+qigong%5bTitle/Abstract%5d+OR+baduanjin%5bTitle/Abstract%5d+OR+exergam*%5bTitle/Abstract%5d+OR+treadmill*%5bTitle/Abstract%5d+OR+stair+climbing%5bTitle/Abstract%5d+OR+endurance+training*%5bTitle/Abstract%5d+OR+athletic*%5bTitle/Abstract%5d+OR+high-intensity+interval+training*%5bTitle/Abstract%5d+OR+HIIT%5bTitle/Abstract%5d&ac=no&sort=relevance) | "exercise*"[Title/Abstract] OR "sport*"[Title/Abstract] OR "training*"[Title/Abstract] OR "aerobic exercis*"[Title/Abstract] OR "plyometric exercis*"[Title/Abstract] OR "resistance training*"[Title/Abstract] OR "strength training*"[Title/Abstract] OR "weight training*"[Title/Abstract] OR "physical activit*"[Title/Abstract] OR "stretch*"[Title/Abstract] OR "muscle stretch*"[Title/Abstract] OR "jogging"[Title/Abstract] OR "walking"[Title/Abstract] OR "ambulation"[Title/Abstract] OR "running"[Title/Abstract] OR "swimming"[Title/Abstract] OR "dancing"[Title/Abstract] OR "cycling"[Title/Abstract] OR "biking"[Title/Abstract] OR "yoga"[Title/Abstract] OR "pilates"[Title/Abstract] OR "tai ji"[Title/Abstract] OR "taijiquan"[Title/Abstract] OR "qi gong"[Title/Abstract] OR "qigong"[Title/Abstract] OR "baduanjin"[Title/Abstract] OR "exergam*"[Title/Abstract] OR "treadmill*"[Title/Abstract] OR "stair climbing"[Title/Abstract] OR "endurance training*"[Title/Abstract] OR "athletic*"[Title/Abstract] OR "high intensity interval training*"[Title/Abstract] OR "HIIT"[Title/Abstract] |
| #3 | [1,648,442](https://pubmed.ncbi.nlm.nih.gov/?term=(exercise%5bMeSH+Terms%5d)+OR+(exercise*%5bTitle/Abstract%5d+OR+sport*%5bTitle/Abstract%5d+OR+training*%5bTitle/Abstract%5d+OR+aerobic+exercis*%5bTitle/Abstract%5d+OR+plyometric+exercis*%5bTitle/Abstract%5d+OR+resistance+training*%5bTitle/Abstract%5d+OR+strength+training*%5bTitle/Abstract%5d+OR+weight+training*%5bTitle/Abstract%5d+OR+physical+activit*%5bTitle/Abstract%5d+OR+stretch*%5bTitle/Abstract%5d+OR+muscle+stretch*%5bTitle/Abstract%5d+OR+jogging%5bTitle/Abstract%5d+OR+walking%5bTitle/Abstract%5d+OR+ambulation%5bTitle/Abstract%5d+OR+running%5bTitle/Abstract%5d+OR+swimming%5bTitle/Abstract%5d+OR+dancing%5bTitle/Abstract%5d+OR+cycling%5bTitle/Abstract%5d+OR+biking%5bTitle/Abstract%5d+OR+yoga%5bTitle/Abstract%5d+OR+pilates%5bTitle/Abstract%5d+OR+tai+ji%5bTitle/Abstract%5d+OR+taijiquan%5bTitle/Abstract%5d+OR+qi+gong%5bTitle/Abstract%5d+OR+qigong%5bTitle/Abstract%5d+OR+baduanjin%5bTitle/Abstract%5d+OR+exergam*%5bTitle/Abstract%5d+OR+treadmill*%5bTitle/Abstract%5d+OR+stair+climbing%5bTitle/Abstract%5d+OR+endurance+training*%5bTitle/Abstract%5d+OR+athletic*%5bTitle/Abstract%5d+OR+high-intensity+interval+training*%5bTitle/Abstract%5d+OR+HIIT%5bTitle/Abstract%5d)&ac=no&sort=relevance) | #1 OR #2 |
| #4 | [302,830](https://pubmed.ncbi.nlm.nih.gov/?term=overweight%5bMeSH+Terms%5d&ac=no&sort=relevance) | "overweight"[MeSH Terms] |
| #5 | [290,675](https://pubmed.ncbi.nlm.nih.gov/?term=obesity%5bMeSH+Terms%5d&ac=no&sort=relevance) | "obesity"[MeSH Terms] |
| #6 | [485,229](https://pubmed.ncbi.nlm.nih.gov/?term=overweight%5bTitle/Abstract%5d+OR+obes*%5bTitle/Abstract%5d+OR+excess+weight%5bTitle/Abstract%5d+OR+excess+body+weight%5bTitle/Abstract%5d+OR+high+body+mass+index%5bTitle/Abstract%5d+OR+high+BMI%5bTitle/Abstract%5d&ac=no&sort=relevance) | "overweight"[Title/Abstract] OR "obes*"[Title/Abstract] OR "excess weight"[Title/Abstract] OR "excess body weight"[Title/Abstract] OR "high body mass index"[Title/Abstract] OR "high bmi"[Title/Abstract] |
| #7 | [537,167](https://pubmed.ncbi.nlm.nih.gov/?term=((overweight%5bMeSH+Terms%5d)+OR+(obesity%5bMeSH+Terms%5d))+OR+(overweight%5bTitle/Abstract%5d+OR+obes*%5bTitle/Abstract%5d+OR+excess+weight%5bTitle/Abstract%5d+OR+excess+body+weight%5bTitle/Abstract%5d+OR+high+body+mass+index%5bTitle/Abstract%5d+OR+high+BMI%5bTitle/Abstract%5d)&ac=no&sort=relevance) | #4 OR #5 OR #6 |
| #8 | [2,305,154](https://pubmed.ncbi.nlm.nih.gov/?term=child%5bMeSH+Terms%5d&ac=no&sort=relevance) | "child"[MeSH Terms] |
| #9 | [2,375,113](https://pubmed.ncbi.nlm.nih.gov/?term=adolescent%5bMeSH+Terms%5d&ac=no&sort=relevance) | "adolescent"[MeSH Terms] |
| #10 | [2,432,631](https://pubmed.ncbi.nlm.nih.gov/?term=child*%5bTitle/Abstract%5d+OR+adolescen*%5bTitle/Abstract%5d+OR+youth*%5bTitle/Abstract%5d+OR+teen*%5bTitle/Abstract%5d+OR+pediatric*%5bTitle/Abstract%5d+OR+paediatric*%5bTitle/Abstract%5d+OR+school-age*%5bTitle/Abstract%5d+OR+young+people%5bTitle/Abstract%5d+OR+young+person*%5bTitle/Abstract%5d&ac=no&sort=relevance) | "child*"[Title/Abstract] OR "adolescen*"[Title/Abstract] OR "youth*"[Title/Abstract] OR "teen*"[Title/Abstract] OR "pediatric*"[Title/Abstract] OR "paediatric*"[Title/Abstract] OR "school age*"[Title/Abstract] OR "young people"[Title/Abstract] OR "young person*"[Title/Abstract] |
| #11 | [4,393,937](https://pubmed.ncbi.nlm.nih.gov/?term=((child*%5bTitle/Abstract%5d+OR+adolescen*%5bTitle/Abstract%5d+OR+youth*%5bTitle/Abstract%5d+OR+teen*%5bTitle/Abstract%5d+OR+pediatric*%5bTitle/Abstract%5d+OR+paediatric*%5bTitle/Abstract%5d+OR+school-age*%5bTitle/Abstract%5d+OR+young+people%5bTitle/Abstract%5d+OR+young+person*%5bTitle/Abstract%5d)+OR+(adolescent%5bMeSH+Terms%5d))+OR+(child%5bMeSH+Terms%5d)&ac=no&sort=relevance) | #8 OR #9 OR #10 |
| #12 | [23,296](https://pubmed.ncbi.nlm.nih.gov/?term=executive+function%5bMeSH+Terms%5d&sort=) | "executive function"[MeSH Terms] |
| #13 | [311,317](https://pubmed.ncbi.nlm.nih.gov/?term=inhibitory+control%5bTitle/Abstract%5d+OR+response+inhibition%5bTitle/Abstract%5d+OR+behavioral+inhibition%5bTitle/Abstract%5d+OR+behavioural+inhibition%5bTitle/Abstract%5d+OR+cognitive+inhibition%5bTitle/Abstract%5d+OR+interference+control%5bTitle/Abstract%5d+OR+impulse+control%5bTitle/Abstract%5d+OR+impulsiv*%5bTitle/Abstract%5d+OR+self-control%5bTitle/Abstract%5d+OR+self+control%5bTitle/Abstract%5d+OR+executive+function*%5bTitle/Abstract%5d+OR+executive+control*%5bTitle/Abstract%5d+OR+executive+functioning%5bTitle/Abstract%5d+OR+cognitive+function*%5bTitle/Abstract%5d+OR+cognitive+control*%5bTitle/Abstract%5d+OR+cognitive+abiliti*%5bTitle/Abstract%5d+OR+cognition%5bTitle/Abstract%5d&ac=no&sort=relevance) | "inhibitory control"[Title/Abstract] OR "response inhibition"[Title/Abstract] OR "behavioral inhibition"[Title/Abstract] OR "behavioural inhibition"[Title/Abstract] OR "cognitive inhibition"[Title/Abstract] OR "interference control"[Title/Abstract] OR "impulse control"[Title/Abstract] OR "impulsiv*"[Title/Abstract] OR "self-control"[Title/Abstract] OR "self-control"[Title/Abstract] OR "executive function*"[Title/Abstract] OR "executive control*"[Title/Abstract] OR "executive functioning"[Title/Abstract] OR "cognitive function*"[Title/Abstract] OR "cognitive control*"[Title/Abstract] OR "cognitive abiliti*"[Title/Abstract] OR "cognition"[Title/Abstract] |
| #14 | [314,649](https://pubmed.ncbi.nlm.nih.gov/?term=(executive+function%5bMeSH+Terms%5d)+OR+(inhibitory+control%5bTitle/Abstract%5d+OR+response+inhibition%5bTitle/Abstract%5d+OR+behavioral+inhibition%5bTitle/Abstract%5d+OR+behavioural+inhibition%5bTitle/Abstract%5d+OR+cognitive+inhibition%5bTitle/Abstract%5d+OR+interference+control%5bTitle/Abstract%5d+OR+impulse+control%5bTitle/Abstract%5d+OR+impulsiv*%5bTitle/Abstract%5d+OR+self-control%5bTitle/Abstract%5d+OR+self+control%5bTitle/Abstract%5d+OR+executive+function*%5bTitle/Abstract%5d+OR+executive+control*%5bTitle/Abstract%5d+OR+executive+functioning%5bTitle/Abstract%5d+OR+cognitive+function*%5bTitle/Abstract%5d+OR+cognitive+control*%5bTitle/Abstract%5d+OR+cognitive+abiliti*%5bTitle/Abstract%5d+OR+cognition%5bTitle/Abstract%5d)&ac=no&sort=relevance) | #12 OR #13 |
| #15 | 401 | #3 AND #7 AND #11 AND #14 |

**Table S3** Web of Science

| Step | Amount | Search string |
| --- | --- | --- |
| #1 | [5,737,610](https://www.webofscience.com/wos/woscc/summary/04e1d860-25cd-4a65-b249-ab624a5af4c7-019516e8aa/relevance/1) | TS=(exercise* OR sport* OR training* OR aerobic exercis* OR plyometric exercis* OR resistance training* OR strength training* OR weight training* OR physical activit* OR stretch* OR muscle stretch* OR jogging OR walking OR ambulation OR running OR swimming OR dancing OR cycling OR biking OR yoga OR pilates OR tai ji OR taijiquan OR qi gong OR qigong OR baduanjin OR exergam* OR treadmill* OR stair climbing OR endurance training* OR athletic* OR high-intensity interval training* OR HIIT) |
| #2 | [798,421](https://www.webofscience.com/wos/woscc/summary/0b3a300a-b6db-422c-ad6d-884589c49d46-019516e9e9/relevance/1) | TS=(overweight OR obes* OR excess weight OR excess body weight OR high body mass index OR high BMI) |
| #3 | [3,235,637](https://www.webofscience.com/wos/woscc/summary/76c3cac7-0ed2-4d39-983b-62836840857e-019516eb39/relevance/1) | TS=(child* OR adolescen* OR youth* OR teen* OR pediatric* OR paediatric* OR school-age* OR young people OR young person*) |
| #4 | [1,708,424](https://www.webofscience.com/wos/woscc/summary/4a0d8e65-18c1-493c-8a1c-2f90e6d4547a-019516ecf8/relevance/1) | TS=(inhibitory control OR response inhibition OR behavioral inhibition OR behavioural inhibition OR cognitive inhibition OR interference control OR impulse control OR impulsiv* OR self-control OR self control OR executive function* OR executive control* OR executive functioning OR cognitive function* OR cognitive control* OR cognitive abiliti* OR cognition) |
| #5 | [3,557](https://www.webofscience.com/wos/woscc/summary/14cbc4e7-32a1-444b-be67-1d7615db2447-019516f1cb/relevance/1) | #1 AND #2 AND #3 AND #4 |

**Table S4** Cochrane library

| Step | Amount | Search string |
| --- | --- | --- |
| #1 | 41,880 | MeSH descriptor: [Exercise] explode all trees |
| #2 | 416,477 | (exercise* OR sport* OR training* OR aerobic exercis* OR plyometric exercis* OR resistance training* OR strength training* OR weight training* OR physical activit* OR stretch* OR muscle stretch* OR jogging OR walking OR ambulation OR running OR swimming OR dancing OR cycling OR biking OR yoga OR pilates OR tai ji OR taijiquan OR qi gong OR qigong OR baduanjin OR exergam* OR treadmill* OR stair climbing OR endurance training* OR athletic* OR high-intensity interval training* OR HIIT):ti,ab,kw |
| #3 | 416,493 | #1 OR #2 |
| #4 | 26,603 | MeSH descriptor: [Overweight] explode all trees |
| #5 | 22,798 | MeSH descriptor: [Obesity] explode all trees |
| #6 | 87,107 | (overweight OR obes* OR excess weight OR excess body weight OR high body mass index OR high BMI):ti,ab,kw |
| #7 | 87,207 | #4 OR #5 OR #6 |
| #8 | 84,192 | MeSH descriptor: [Child] explode all trees |
| #9 | 139,680 | MeSH descriptor: [Adolescent] explode all trees |
| #10 | 365,017 | (child* OR adolescen* OR youth* OR teen* OR pediatric* OR paediatric* OR school-age* OR young people OR young person*):ti,ab,kw |
| #11 | 365,017 | #8 OR #9 OR #10 |
| #12 | 2,024 | MeSH descriptor: [Executive Function] explode all trees |
| #13 | 277,098 | (inhibitory control OR response inhibition OR behavioral inhibition OR behavioural inhibition OR cognitive inhibition OR interference control OR impulse control OR impulsiv* OR self-control OR self control OR executive function* OR executive control* OR executive functioning OR cognitive function* OR cognitive control* OR cognitive abiliti* OR cognition):ti,ab,kw |
| #14 | 277,098 | #12 OR #13 |
| #15 | 2,018 | #3 AND #7 AND #11 AND #14 |

**Table S5** Embase

| Step | Amount | Search string |
| --- | --- | --- |
| #1 | 562,556 | 'exercise'/exp |
| #2 | [2,066,722](http://www--embase--com--https.embase.shd1rmyy.lwnote.com:50001/) | 'exercise':ab,ti,kw OR 'exercises':ab,ti,kw OR 'sport':ab,ti,kw OR 'sports':ab,ti,kw OR 'training':ab,ti,kw OR 'trainings':ab,ti,kw OR 'aerobic exercise':ab,ti,kw OR 'plyometric exercise':ab,ti,kw OR 'resistance training':ab,ti,kw OR 'strength training':ab,ti,kw OR 'muscle stretching exercises':ab,ti,kw OR 'physical activity':ab,ti,kw OR 'physical activities':ab,ti,kw OR 'jogging':ab,ti,kw OR 'walking':ab,ti,kw OR 'ambulation':ab,ti,kw OR 'tai ji':ab,ti,kw OR 'taijiquan':ab,ti,kw OR 'yoga':ab,ti,kw OR 'swimming':ab,ti,kw OR 'dancing':ab,ti,kw OR 'cycling':ab,ti,kw OR 'pilates':ab,ti,kw OR 'stretching':ab,ti,kw OR 'biking':ab,ti,kw OR 'weight training':ab,ti,kw OR 'running':ab,ti,kw OR 'exergaming':ab,ti,kw OR 'treadmill':ab,ti,kw OR 'qi gong':ab,ti,kw OR 'baduanjin':ab,ti,kw OR 'stair climbing':ab,ti,kw OR 'endurance training':ab,ti,kw OR 'athletics':ab,ti,kw OR 'high-intensity interval training':ab,ti,kw OR 'hiit':ab,ti,kw |
| #3 | 2,179,158 | #1 or #2 |
| #4 | 818,634 | 'obesity'/exp |
| #5 | [746,950](http://www--embase--com--https.embase.shd1rmyy.lwnote.com:50001/) | 'overweight':ab,ti,kw OR 'obesity':ab,ti,kw OR 'obese':ab,ti,kw OR 'excess weight':ab,ti,kw OR 'excess body weight':ab,ti,kw OR 'high body mass index':ab,ti,kw OR 'high bmi':ab,ti,kw |
| #6 | 979,017 | #4 OR #5 |
| #7 | 3,816,573 | 'child'/exp |
| #8 | 2,187,540 | 'adolescent'/exp |
| #9 | [3,007,188](http://www--embase--com--https.embase.shd1rmyy.lwnote.com:50001/) | 'child':ab,ti,kw OR 'children':ab,ti,kw OR 'adolescent':ab,ti,kw OR 'adolescents':ab,ti,kw OR 'youth':ab,ti,kw OR 'youths':ab,ti,kw OR 'teen':ab,ti,kw OR 'teens':ab,ti,kw OR 'teenager':ab,ti,kw OR 'teenagers':ab,ti,kw OR 'pediatric':ab,ti,kw OR 'paediatric':ab,ti,kw OR 'school-age':ab,ti,kw OR 'school aged':ab,ti,kw OR 'young people':ab,ti,kw OR 'young person':ab,ti,kw OR 'young persons':ab,ti,kw |
| #10 | [5,554,873](http://www--embase--com--https.embase.shd1rmyy.lwnote.com:50001/) | #7 OR #8 OR #9 |
| #11 | 128,554 | 'executive function'/exp |
| #12 | [432,082](http://www--embase--com--https.embase.shd1rmyy.lwnote.com:50001/) | 'inhibitory control':ab,ti,kw OR 'response inhibition':ab,ti,kw OR 'behavioral inhibition':ab,ti,kw OR 'behavioural inhibition':ab,ti,kw OR 'cognitive inhibition':ab,ti,kw OR 'interference control':ab,ti,kw OR 'impulse control':ab,ti,kw OR 'impulsivity':ab,ti,kw OR 'impulsive':ab,ti,kw OR 'self-control':ab,ti,kw OR 'self control':ab,ti,kw OR 'executive function':ab,ti,kw OR 'executive functions':ab,ti,kw OR 'executive control':ab,ti,kw OR 'executive controls':ab,ti,kw OR 'executive functioning':ab,ti,kw OR 'cognitive function':ab,ti,kw OR 'cognitive functions':ab,ti,kw OR 'cognitive control':ab,ti,kw OR 'cognitive controls':ab,ti,kw OR 'cognitive ability':ab,ti,kw OR 'cognitive abilities':ab,ti,kw OR 'cognition':ab,ti,kw |
| #13 | 477,842 | #11 OR #12 |
| #14 | 660 | #3 AND #6 AND #10 AND #13 |

**Table S6** CNKI

| Step | Amount | Search string |
| --- | --- | --- |
| #1 | 4,630,400 | 运动 + 体育活动 + 体育运动 + 训练 + 爆发力训练 + 有氧运动 + 阻力训练 + 力量训练 + 肌肉拉伸运动 + 身体活动 + 慢跑 + 步行 + 太极 + 太极拳 + 瑜伽 + 游泳 + 跳舞 + 骑行 + 普拉提 + 举重训练 + 跑步 + 运动游戏 + 跑步机 + 气功 + 八段锦 + 爬楼梯 + 耐力训练 + 田径 + 高强度间歇训练 |
| #2 | 838,000 | 抑制 + 抑制控制 + 反应抑制 + 行为抑制 + 冲动控制 + 自我控制 + 认知抑制 + 干扰抑制 + 认知功能 + 认知 + 认知益处 + 认知表现 + 执行功能 |
| #3 | 73,000 | 超重 + 肥胖 |
| #4 | 620,900 | 儿童 + 青少年 |
| #5 | 87 | #1 AND #2 AND #3 AND #4 |

## 1.3 Selection Criteria

The studies were included according to the following criteria:

(1) Participants: All included studies enrolled participants diagnosed as overweight or obese. Because diagnostic criteria for overweight and obesity vary across countries and regions, no single standard was imposed. Studies were considered eligible if they explicitly reported that participants met the World Health Organization (WHO) criteria or region- or country-specific definitions (e.g., Asian or European standards). When diagnostic criteria were not clearly specified but objective measures such as body mass index (BMI) were reported, eligibility was determined through a comprehensive evaluation of the available data[1]. Studies were included if the mean participant age ranged from 6 to 18 years, even when a small proportion of individuals fell outside this range.

(2) Intervention: No restrictions were placed on the type of exercise intervention. Eligible studies were required to include at least one exercise-only intervention group, which could involve one or multiple forms of exercise. To reduce potential confounding, studies combining exercise with non-exercise components—such as dietary modification or pharmacological treatment—were excluded. In studies with multiple intervention arms, only data from exercise-only arms were extracted; studies in which all arms included non-exercise components were excluded. Furthermore, only long-term exercise interventions were considered, and studies examining acute exercise effects were not included[2, 3].

(3) Control group: Control groups were not allowed to receive any exercise intervention to prevent contamination and ensure comparability of effects. Participants in control conditions could maintain their usual activities, including unstructured physical activity or standard educational curricula, and could receive routine rehabilitation or supportive care (e.g., psychological therapy), provided these did not involve structured or organized exercise.

(4) Outcome: The primary outcome was inhibitory control. Studies evaluating inhibitory control in children and adolescents with overweight or obesity were eligible, regardless of whether assessments were based on standardized questionnaires or experimental task paradigms[4-8].

(5) Study design: Only experimental studies employing a pre–post design were included, comprising randomized controlled trials (RCTs) and quasi-experimental studies.

(6) Studies were published in English or Chinese.

(7) Studies were published in peer-reviewed journals.

(8) Studies reported sufficient data to enable the calculation of effect sizes.

(9) Studies involving human participants.

**1.4 Data Extraction**

Data extraction was performed independently by two reviewers (PW and DL) using a predesigned, standardized extraction form. Inter-rater agreement was high (95.08%). Discrepancies were resolved through discussion between the two reviewers; when consensus could not be reached, a third reviewer (JH) was consulted to make a final determination. Extracted data included basic study identifiers (authors and year of publication) and moderator variables. Moderator variables were defined according to the evidence-based PICO framework and categorized as Population, Intervention, Control, and Outcome.

**1.4.1 Population**

**Age**, treated as a categorical variable, was defined as the mean age of participants in the intervention groups. Inhibitory control exhibits distinct developmental trajectories across childhood and adolescence. During the school-age period (6–11 years), inhibitory control improves rapidly, reflecting ongoing maturation of the prefrontal cortex and large-scale control networks, and is characterized by high neural plasticity and notable behavioral gains[9, 10]. In adolescence, inhibitory control continues to develop, but the rate of improvement slows and gradually approaches maturity, reaching near-adult levels around 14–15 years of age[11, 12]. Based on these developmental differences, participants were categorized as children (6–11 years) or adolescents (12–18 years).

**1.4.2 Intervention**

**Type of motor skills**, defined as a categorical variable, was classified into open and closed motor skills[13, 14]. Open motor skills involve performing movements in unpredictable environments and require continuous adaptation and rapid responses to environmental changes. In contrast, closed motor skills are performed in stable and predictable environments, allowing movements to be planned in advance. Cognitive benefits derived from exercise may vary by skill type[15].

**Training frequency**, also treated as a categorical variable, was defined as the number of training sessions per week. As all included studies reported weekly training frequency, and observed values were limited to 2, 3, 5, or 10 sessions per week, frequency was categorized into four corresponding groups.

**Training intensity** was categorized as moderate physical activity (MPA), moderate-to-vigorous physical activity (MVPA), or vigorous physical activity (VPA). When studies explicitly reported exercise intensity using terms such as “moderate,” “vigorous,” or “MVPA,” classifications followed the authors’ descriptions. When such terminology was absent but objective physiological measures or subjective ratings of perceived exertion were provided, intensity was classified according to the guidelines of the American College of Sports Medicine (ACSM)[16]. Under these guidelines, moderate intensity corresponds to 64%–76% of maximum heart rate (HRmax), 40%–59% of heart rate reserve (HRR), 46%–63% of maximal oxygen uptake (VO₂max), or a rating of perceived exertion (RPE) of 12–13. Vigorous intensity corresponds to 77%–95% of HRmax, 60%–89% of HRR, 64%–90% of VO₂max, or an RPE of 14–17. MVPA was defined when any indicator spanned both moderate and vigorous intensity ranges. For example, an intervention reporting an HRmax range of 70%–85% was classified as MVPA because the lower bound fell within the moderate range and the upper bound within the vigorous range. When only mean heart rate during the intervention was reported, the proportion of HRmax was estimated using established methods. For instance, a mean heart rate of 138 beats per minute in a 10-year-old child was estimated to correspond to approximately 70% of HRmax[17]. Maximum heart rate was calculated using the formula: HRmax = 208 − 0.7 × age[18].

**1.4.3 Control**

**Control groups**, treated as a categorical variable, were classified as active or passive. Active control groups continued usual care (e.g., compulsory education or outpatient services) and received additional non-exercise interventions during the study period, such as reading activities, cognitive games, or psychological therapy. Control participants who continued to attend regular school-based physical education classes were also classified as active controls. Passive control groups received no additional interventions beyond usual care and completed only pre- and post-intervention assessments.

**1.4.4 Outcome**

**Task performance metrics** indicators comprised quantitative or qualitative measures used to assess the quality, speed, or accuracy of performance on inhibitory control–related tasks or questionnaires. Based on their characteristics, indicators were classified into three domains: time, accuracy, and score. Time-based measures included mean reaction time, overall reaction time, and total task completion time. Reaction time was defined as the interval between stimulus presentation and the participant’s response (e.g., pressing a computer key). Accuracy reflected the proportion of correct responses relative to the total number of trials. Scores referred to quantitative values obtained from standardized questionnaires.

### 1.5 Statistical Analysis

Effect sizes and variances are calculated in Excel, consistently with Aksayli et al[19]. Subsequent statistical analyses were conducted using the metafor package in R Version 4.4.0 (R Core Team 2024)[20].

### 1.5.1 Effect size Calculation

Hedges’g was used as the effect size metric in this study[21], calculated as: $g=\frac{\left( M_{e\_post}-M_{e\_pre} \right)-(M_{c\_post}-M_{c\_pre})}{\mathrm{SD}_{pooled\_pre}}$× (1- $\frac{3}{4\times N-9}$), where Me_post and M_c_post_ represent the post-intervention means of the experimental and control groups, respectively, M_e_pre_ and M_c_pre_ are the corresponding pre-intervention means, SD_pooled_pre_ is the pooled pre-test standard deviation, and N is the total sample size. This approach is appropriate for calculating standardized mean differences in repeated-measures intervention studies[22]. Positive values of g indicate greater “benefit” in the intervention group relative to the control group, where “benefit” refers to improvement in inhibitory control. To ensure consistency in effect size directionality (i.e., positive g always reflecting improvements in inhibitory control), effect sizes derived from reverse-scored paradigms or questionnaires were multiplied by −1.

In repeated-measures designs, accurate estimation of sampling variance typically requires the pre–post correlation[23], which was often not reported in the included studies. Following Aksayli et al., sampling variance was approximated using the formula: $\mathrm{Var}_{g}=(\frac{N}{N_{e}\times N_{c}}+\frac{d^{2}}{2\times N})\times{(1- \frac{3}{4\times N-9})}^{2}$, where N_e_ and N_c_ are the sample sizes of the experimental and control groups, respectively, N=N_e_+N_c_, and d is the uncorrected standardized mean difference.

### 1.5.2 Meta-analysis

In this dataset, some studies reported multiple outcome measures, leading to dependency among effect sizes. To retain all effect sizes while accounting for this dependency and maximizing statistical power, multilevel meta-analysis was used instead of multivariate meta-analysis[24-26]. This choice was motivated by the fact that most included studies did not report correlations among outcomes required for multivariate meta-analysis. Multilevel models allow the estimation of variance at three levels: sampling variance (level 1), within-study variance among effect sizes (level 2), and between-study variance (level 3).

First, overall effect sizes were estimated using a three-level random-effects model. Following prior methodological recommendations[24, 27], the rma.mv function was applied with test = “t” and restricted maximum likelihood (REML) estimation. Given heterogeneity in participant characteristics, interventions, and outcome measures across studies, and the aim to generalize findings to a broader population, a random-effects framework was adopted[21].

Second, an influence analysis was conducted to identify effect sizes potentially exerting a substantial impact on the overall results and to assess their influence[20]. In this study, an effect size was considered influential if its removal led to notable changes in the overall estimate[20]. Meta-analysis models were refitted both including and excluding these influential effect sizes to evaluate result robustness.

Third, Cochran’s Q statistic was used to test overall heterogeneity, calculated as the weighted sum of squared deviations of individual effect sizes from the overall effect size[28]. I² was computed for each of the three levels to estimate the proportion of variance attributable to each level[27]. Additionally, two likelihood ratio tests were conducted to compare model fit and determine whether the three-level model offered improvements over a two-level model: first, the level-2 variance was set to zero to fit a two-level model (levels 1 and 3) for comparison; second, the level-3 variance was set to zero and compared again with the full model[27].

Fourth, subgroup analyses were performed using a three-level mixed-effects meta-analysis model[21]. The omnibus Q test was applied to determine whether differences among subgroups exceeded what would be expected by sampling error alone[24].

Fifth, three-level meta-regression was conducted to examine the moderating effect of baseline body mass index (BMI) on intervention outcomes[25]. Baseline BMI was included as a continuous moderator to assess its linear relationship with effect size, while retaining the three-level structure to account for sampling variance, within-study variability, and between-study variability.

Sixth, to evaluate potential nonlinear relationships between continuous variables and effect sizes, cubic spline meta-regression models were fitted, with study ID included as a random factor to account for dependency among effect sizes. Training session (continuous variable) was defined as the total number of training sessions, while training duration (continuous variable) was calculated as the product of session number and duration per session, reflecting total participant training time. Cubic spline linear regression models with three, four, and five knots were fitted and compared using likelihood ratio tests to identify the best-fitting model[29].

Finally, publication bias was assessed using funnel plots[30], Egger’s regression test[31], and the trim-and-fill method[32]. Since funnel plots and trim-and-fill have not yet been adapted for three-level models, these methods were applied based on a two-level model, treating all effect sizes as independent[32, 33]. Egger’s regression test results were reported for both two- and three-level models. In the three-level model, the predictor was set to 2√N, where N is the total sample size of each study, following previous recommendations[32, 33].

**2 Supplementary Result**

### 2.1 Characteristics of the studies

**Table S7** Characteristics of studies and effect sizes included in the meta-analysis of inhibitory control

| **Study** | **Study design** | **N** | **Age** | **BMI** | **Control**  **group** | **Training**  **type** | **Training**  **intensity** | **Per week** | **Sessions** | **Duration** | **Task performance metrics** | **Assessment** | **g** |
| --- | --- | --- | --- | --- | --- | --- | --- | --- | --- | --- | --- | --- | --- |
| (Shang et al., 2022)-1 | RCT | 52 | NR | NR | active | open | NR | three | 24 | 1320 | time | Flanker task | 0.5804 |
| (Yuan et al., 2025)-1 | RCT | 40 | 13.67 | 22.93 | active | open | MPA | three | 36 | 1440 | score | BRIEF-P | 1.0564 |
| (Yuan et al., 2025)-2 | RCT | 40 | 13.67 | 22.93 | active | closed | MPA | three | 36 | 1440 | score | BRIEF-P | 0.3428 |
| (Krafft et al., 2014)-1 | RCT | 40 | 9.8 | NR | active | closed | VPA | five | 138 | 5520 | ACC | Antisaccade | 0.0414 |
| (Krafft et al., 2014)-2 | RCT | 40 | 9.8 | NR | active | closed | VPA | five | 138 | 5520 | time | Antisaccade | -0.0627 |
| (Krafft et al., 2014)-3 | RCT | 40 | 9.8 | NR | active | closed | VPA | five | 138 | 5520 | time | Antisaccade | 0.7345 |
| (Krafft et al., 2014)-4 | RCT | 40 | 9.8 | NR | active | closed | VPA | five | 138 | 5520 | ACC | Antisaccade | -0.4646 |
| (Krafft et al., 2014)-5 | RCT | 40 | 9.8 | NR | active | closed | VPA | five | 138 | 5520 | ACC | Antisaccade | 0.8632 |
| (Krafft et al., 2014)-6 | RCT | 39 | 9.8 | NR | active | closed | VPA | five | 138 | 5520 | ACC | Flanker task | 0.1979 |
| (Krafft et al., 2014)-7 | RCT | 39 | 9.8 | NR | active | closed | VPA | five | 138 | 5520 | time | Flanker task | 0.5902 |
| (Krafft et al., 2014)-8 | RCT | 39 | 9.8 | NR | active | closed | VPA | five | 138 | 5520 | ACC | Flanker task | 0.3620 |
| (Krafft et al., 2014)-9 | RCT | 39 | 9.8 | NR | active | closed | VPA | five | 138 | 5520 | time | Flanker task | 0.2328 |
| (Krafft et al., 2014)-10 | RCT | 39 | 9.8 | NR | active | closed | VPA | five | 138 | 5520 | ACC | Flanker task | -0.3505 |
| (Chou et al., 2023)-1 | RCT | 50 | 11.23 | 20.76 | active | open | MVPA | five | 50 | 2500 | time | Stroop test | 1.2945 |
| (Chou et al., 2023)-2 | RCT | 50 | 11.23 | 20.76 | active | open | MVPA | five | 50 | 2500 | time | Stroop test | 1.0263 |
| (Chou et al., 2023)-3 | RCT | 50 | 11.23 | 20.76 | active | open | MVPA | five | 50 | 2500 | time | Stroop test | 3.0435 |
| (Chou et al., 2023)-4 | RCT | 50 | 11.23 | 20.76 | active | open | MVPA | five | 50 | 2500 | time | Stroop test | 2.3664 |
| (Chou et al., 2023)-5 | RCT | 50 | 11.23 | 20.76 | active | open | MVPA | five | 50 | 2500 | score | Stroop test | 1.2079 |
| (Chou et al., 2023)-6 | RCT | 50 | 11.23 | 20.76 | active | open | MVPA | five | 50 | 2500 | score | Stroop test | 1.0404 |
| (Chou et al., 2023)-7 | RCT | 50 | 11.23 | 20.76 | active | open | MVPA | five | 50 | 1500 | time | Stroop test | 1.2945 |
| (Chou et al., 2023)-8 | RCT | 50 | 11.23 | 20.76 | active | open | MVPA | five | 50 | 1500 | time | Stroop test | 0.2074 |
| (Chou et al., 2023)-9 | RCT | 50 | 11.23 | 20.76 | active | open | MVPA | five | 50 | 1500 | time | Stroop test | 3.4450 |
| (Chou et al., 2023)-10 | RCT | 50 | 11.23 | 20.76 | active | open | MVPA | five | 50 | 1500 | time | Stroop test | 2.0880 |
| (Chou et al., 2023)-11 | RCT | 50 | 11.23 | 20.76 | active | open | MVPA | five | 50 | 1500 | score | Stroop test | 1.1327 |
| (Chou et al., 2023)-12 | RCT | 50 | 11.23 | 20.76 | active | open | MVPA | five | 50 | 1500 | score | Stroop test | 1.0329 |
| (Liu et al., 2018)-1 | RCT | 70 | 14.06 | 27.97 | passive | closed | MPA | two | 24 | 1800 | time | Stroop test | 0.5307 |
| (Liu et al., 2018)-2 | RCT | 70 | 14.06 | 27.97 | passive | closed | MPA | two | 24 | 1800 | time | Stroop test | 0.7119 |
| (Liu et al., 2018)-3 | RCT | 70 | 14.06 | 27.97 | passive | closed | MPA | two | 24 | 1800 | time | Stroop test | 0.6192 |
| (Liu et al., 2018)-4 | RCT | 70 | 14.06 | 27.97 | passive | closed | MPA | two | 24 | 1800 | time | Stroop test | 0.8932 |
| (Chou et al., 2020)-1 | RCT | 84 | 12.19 | 24.84 | active | open | MVPA | three | 24 | 960 | time | Stroop test | -0.2784 |
| (Chou et al., 2020)-2 | RCT | 84 | 12.19 | 24.84 | active | open | MVPA | three | 24 | 960 | time | Stroop test | 0.0000 |
| (Chou et al., 2020)-3 | RCT | 84 | 12.19 | 24.84 | active | open | MVPA | three | 24 | 960 | time | Stroop test | 0.5113 |
| (Chou et al., 2020)-4 | RCT | 84 | 12.19 | 24.84 | active | open | MVPA | three | 24 | 960 | time | Stroop test | 0.4772 |
| (Chou et al., 2020)-5 | RCT | 84 | 12.19 | 24.84 | active | open | MVPA | three | 24 | 960 | time | Stroop test | 0.6381 |
| (Chou et al., 2020)-6 | RCT | 84 | 12.19 | 24.84 | active | open | MVPA | three | 24 | 960 | time | Stroop test | 0.7124 |
| (Logan et al., 2021)-1 | RCT | 103 | 8.75 | 25.34 | passive | open | MVPA | five | 150 | 18000 | ACC | Flanker task | 0.2606 |
| (Logan et al., 2021)-2 | RCT | 103 | 8.75 | 25.34 | passive | open | MVPA | five | 150 | 18000 | ACC | Flanker task | 0.0571 |
| (Logan et al., 2021)-3 | RCT | 103 | 8.75 | 25.34 | passive | open | MVPA | five | 150 | 18000 | time | Flanker task | 0.0277 |
| (Logan et al., 2021)-4 | RCT | 103 | 8.75 | 25.34 | passive | open | MVPA | five | 150 | 18000 | time | Flanker task | -0.0146 |
| (Logan et al., 2021)-5 | RCT | 103 | 8.75 | 25.34 | passive | open | MVPA | five | 150 | 18000 | ACC | Flanker task | 0.3550 |
| (Logan et al., 2021)-6 | RCT | 103 | 8.75 | 25.34 | passive | open | MVPA | five | 150 | 18000 | time | Flanker task | -0.1206 |
| (Mora-Gonzalez et al., 2024)-1 | RCT | 61 | 10 | 26.58 | passive | open | MVPA | five | 100 | 9000 | time | Flanker task | -0.0441 |
| (Mora-Gonzalez et al., 2024)-2 | RCT | 61 | 10 | 26.58 | passive | open | MVPA | five | 100 | 9000 | ACC | Flanker task | -0.4186 |
| (Mora-Gonzalez et al., 2024)-3 | RCT | 61 | 10 | 26.58 | passive | open | MVPA | five | 100 | 9000 | time | Flanker task | 0.0157 |
| (Mora-Gonzalez et al., 2024)-4 | RCT | 61 | 10 | 26.58 | passive | open | MVPA | five | 100 | 9000 | ACC | Flanker task | -0.1438 |
| (Ortega et al., 2022)-1 | RCT | 90 | 10.04 | 26.81 | active | open | MVPA | five | 100 | 9000 | time | Stroop color and word test | 0.0122 |
| (Wang et al., 2024)-1 | QE | 78 | 10.29 | 25.3 | passive | closed | MPA | ten | 40 | 4400 | ACC | GO-NOGO task | 0.2669 |
| (Wang et al., 2024)-2 | QE | 78 | 10.29 | 25.3 | passive | closed | MPA | ten | 40 | 4400 | ACC | GO-NOGO task | 0.3635 |
| (Wang et al., 2024)-3 | QE | 78 | 10.29 | 25.3 | passive | closed | MPA | ten | 40 | 4400 | time | GO-NOGO task | 0.4207 |
| (Wang et al., 2024)-4 | QE | 79 | 10.29 | 25.3 | passive | closed | VPA | ten | 40 | 4400 | ACC | GO-NOGO task | 0.0437 |
| (Wang et al., 2024)-5 | QE | 79 | 10.29 | 25.3 | passive | closed | VPA | ten | 40 | 4400 | ACC | GO-NOGO task | 0.5794 |
| (Wang et al., 2024)-6 | QE | 79 | 10.29 | 25.3 | passive | closed | VPA | ten | 40 | 4400 | time | GO-NOGO task | 0.4407 |
| (Intawachirarat et al., 2025)-1 | RCT | 35 | 10.37 | 24.85 | passive | closed | MPA | three | 30 | 1800 | score | Stroop color and word test | 0.5892 |

Note: “NR” represents “Not Reported”; “Duration” represents “Training Duration,” and it is measured in minutes; “RCT” represents “Randomized controlled trial”; “QE” represents “Quasi-experimental”; “MPA” represents “Moderate physical activity”; “MVPA” represents “Moderate-to-vigorous physical activity”; “VPA” represents “Vigorous physical activity”; “ACC” represents “accuracy”; “open” represents “open motor skills”; “closed” represents “closed motor skills”; “BRIEF-P” represents “Behavior Rating Inventory of Executive Function–Preschool Version”.

| **Table S8** Comparison of Model Fit Superiority (with the Level-2 variance constrained to zero） | | | | | | |  |  |
| --- | --- | --- | --- | --- | --- | --- | --- | --- |
| **Model** | **df** | **AIC** | **BIC** | **AICc** | **LogLik** | **LRT (χ^2^)** | **pval** | **QE** |
| Three-level meta-analysis | 3 | 63.8195 | 69.5556 | 64.3413 | -28.9098 | — | — | 190.8428 |
| Traditional meta-analysis | 2 | 68.4996 | 72.3236 | 68.7549 | -32.2498 | 6.6801 | 0.0097 | 190.8428 |
| Note: AIC = Akaike Information Criterion; BIC = Bayesian Information Criterion; AICc = Corrected AIC; LogLik = Log-Likelihood; LRT = Likelihood Ratio Test. | | | | | | |  |  |

**Table S9** Comparison of Model Fit Superiority (with the Level-3 variance constrained to zero）

| **Model** | **df** | **AIC** | **BIC** | **AICc** | **LogLik** | **LRT (χ^2^)** | **pval** | **QE** |
| --- | --- | --- | --- | --- | --- | --- | --- | --- |
| Three-level meta-analysis | 3 | 63.8195 | 69.5556 | 64.3413 | -28.9098 | — | — | 190.8428 |
| Traditional meta-analysis | 2 | 87.2032 | 91.0273 | 87.4585 | -41.6016 | 25.3837 | < 0.001 | 190.8428 |

Note: AIC = Akaike Information Criterion; BIC = Bayesian Information Criterion; AICc = Corrected AIC; LogLik = Log-Likelihood; LRT = Likelihood Ratio Test.

**Table S10**. Results of Moderator Analyses After Benjamini–Hochberg FDR Correction

| **Moderator** | **QM** | **df** | **p_raw** | **p_adjusted_BH** | **Significant_FDR** |
| --- | --- | --- | --- | --- | --- |
| Age | 1.015358911 | 1 | 0.318675219 | 0.82971386 | No |
| Type of motor skills | 0.092225232 | 1 | 0.762652879 | 0.82971386 | No |
| Training intensity | 0.236495235 | 2 | 0.790323314 | 0.82971386 | No |
| Training frequency | 0.293732373 | 3 | 0.82971386 | 0.82971386 | No |
| Control group | 0.91077193 | 1 | 0.344594233 | 0.82971386 | No |
| Task performance metrics | 0.56858644 | 2 | 0.570093018 | 0.82971386 | No |

Note: QM = omnibus test statistic for moderator analysis; df = degrees of freedom; p_raw = raw p value; p_adjusted_BH = Benjamini–Hochberg false discovery rate adjusted p value; Significant_FDR = statistical significance after false discovery rate correction.

**Table S11** Fit comparison of RCS models for training sessions (3–5 knots)

| **Model (Knots)** | **df** | **AIC** | **BIC** | **LogLik** | **QE** | **Comparison** | **LRT(χ2)** | **p-value** |
| --- | --- | --- | --- | --- | --- | --- | --- | --- |
| 3-Knots | 5 | 80.46 | 90.11 | -35.23 | 152.18 | — | — | — |
| 4-Knots | 6 | 68.93 | 80.52 | -28.46 | 122.42 | 4 vs 3 | 13.53 | < 0.001 |
| 5-Knots | 7 | 72.39 | 85.91 | -29.20 | 123.78 | 5 vs 4 | < 0.001 | > 0.99 |

Note: AIC = Akaike Information Criterion; BIC = Bayesian Information Criterion; AICc = Corrected AIC; LogLik = Log-Likelihood; LRT = Likelihood Ratio Test.

**Table S12** Fit comparison of RCS models for training duration (3–5 knots)

| **Model (Knots)** | **df** | **AIC** | **BIC** | **LogLik** | **QE** | **Comparison** | **LRT(χ2)** | **p-value** |
| --- | --- | --- | --- | --- | --- | --- | --- | --- |
| 3-Knots | 5 | 74.88 | 84.54 | -32.44 | 136.95 | — | — | — |
| 4-Knots | 6 | 63.66 | 75.25 | -25.83 | 107.85 | 4 vs 3 | 13.22 | < 0.001 |
| 5-Knots | 7 | 54.91 | 68.43 | -20.45 | 89.27 | 5 vs 4 | 10.75 | 0.001 |

Note: AIC = Akaike Information Criterion; BIC = Bayesian Information Criterion; AICc = Corrected AIC; LogLik = Log-Likelihood; LRT = Likelihood Ratio Test.

**Table S13** GRADE level of evidence for this study’s findings

| **Outcome** | **Total N** | **k** | **Quality assessment** | | | | | **Hedge’s g(95%CI)** | **Evidence Quality*** |
| --- | --- | --- | --- | --- | --- | --- | --- | --- | --- |
|  |  |  | **Study limitations** | **Consistency** | **Directness** | **Precision** | **Publication bias** |  |  |
| Overall | 787 | 51 | ↓ 1 level^1)^ | ↓ 1 level^2)^ | No down | No down | ↓ 1 level^5)^ | 0.41 [0.16, 0.67] | Very low |
| Children | 521 | 38 | ↓ 1 level^1)^ | ↓ 1 level^2)^ | No down | No down | NA | 0.34 [0.00, 0.67] | Low |
| Adolescents | 214 | 12 | ↓ 1 level^1)^ | No down | No down | ↓ 1 level^4)^ | NA | 0.56 [0.05, 1.07] | Low |
| Open | 505 | 29 | ↓ 1 level^1)^ | ↓ 1 level^2)^ | No down | No down | NA | 0.44 [0.11, 0.78] | Low |
| Closed | 302 | 22 | ↓ 1 level^1)^ | No down | No down | ↓ 2 level^4)^ | NA | 0.37 [-0.03, 0.77] | Very low |
| MI | 243 | 10 | ↓ 1 level^1)^ | No down | No down | ↓ 1 level^4)^ | NA | 0.52 [0.07, 0.96] | Low |
| MVI | 413 | 27 | ↓ 1 level^1)^ | ↓ 1 level^2)^ | No down | ↓ 1 level^4)^ | NA | 0.33 [-0.06, 0.72] | Very low |
| VI | 119 | 13 | ↓ 1 level^1)^ | No down | No down | ↓ 2 level^4)^ | NA | 0.41 [-0.10, 0.93] | Very low |
| Week 2 | 70 | 4 | ↓ 1 level^1)^ | No down | No down | ↓ 2 level^4)^ | NA | 0.69 [-0.23, 1.61] | Very low |
| Week 3 | 231 | 10 | ↓ 1 level^1)^ | No down | No down | ↓ 1 level^4)^ | NA | 0.53 [0.01, 1.05] | Low |
| Week 5 | 369 | 21 | ↓ 1 level^1)^ | ↓ 1 level^2)^ | No down | ↓ 2 level^4)^ | NA | 0.30 [-0.11, 0.71] | Very low |
| Week 10 | 117 | 6 | ↓ 1 level^1)^ | No down | No down | ↓ 2 level^4)^ | NA | 0.35 [-0.55, 1.25] | Very low |
| Active | 401 | 30 | ↓ 1 level^1)^ | No down | No down | ↓ 1 level^4)^ | NA | 0.53 [0.18, 0.87] | Low |
| Passive | 386 | 21 | ↓ 1 level^1)^ | ↓ 1 level^2)^ | No down | ↓ 2 level^4)^ | NA | 0.29 [-0.09, 0.66] | Very low |
| Time | 692 | 29 | ↓ 1 level^1)^ | No down | No down | No down | NA | 0.44 [0.18, 0.70] | Moderate |
| ACC | 321 | 15 | ↓ 1 level^1)^ | No down | No down | ↓ 1 level^4)^ | NA | 0.30 [-0.02, 0.63] | Very low |
| Score | 170 | 7 | ↓ 1 level^1)^ | No down | No down | ↓ 1 level^4)^ | NA | 0.48 [0.06, 0.90] | Very low |
| *GRADE: Quality of Clinical Evidence and Recommendation Levels:  High: The research team has strong confidence in the estimated effect size;  Moderate: The research team has moderate confidence in the estimated effect size;  Low: The research team has limited confidence in the estimated effect size;  Very low: The research team has very limited confidence in the estimated effect size. | | | | | | | | | |

Note: 1) More than two-thirds of the studies were rated as having a moderate risk of bias, so the level is downgraded by one step; 2) There was substantial heterogeneity among the included studies, so the level was downgraded by one step; ↓ 1 level4) The included studies have relatively small sample sizes; ↓ 2 level4) In addition to small sample sizes, the 95% credible interval also includes the null value; 5) There is a high possibility of publication bias, so the level is downgraded by one step; NA: As fewer than ten studies were included, this item was not evaluated.

**2.2 Effect of exercise intervention on inhibitory control**

**Figure S1**


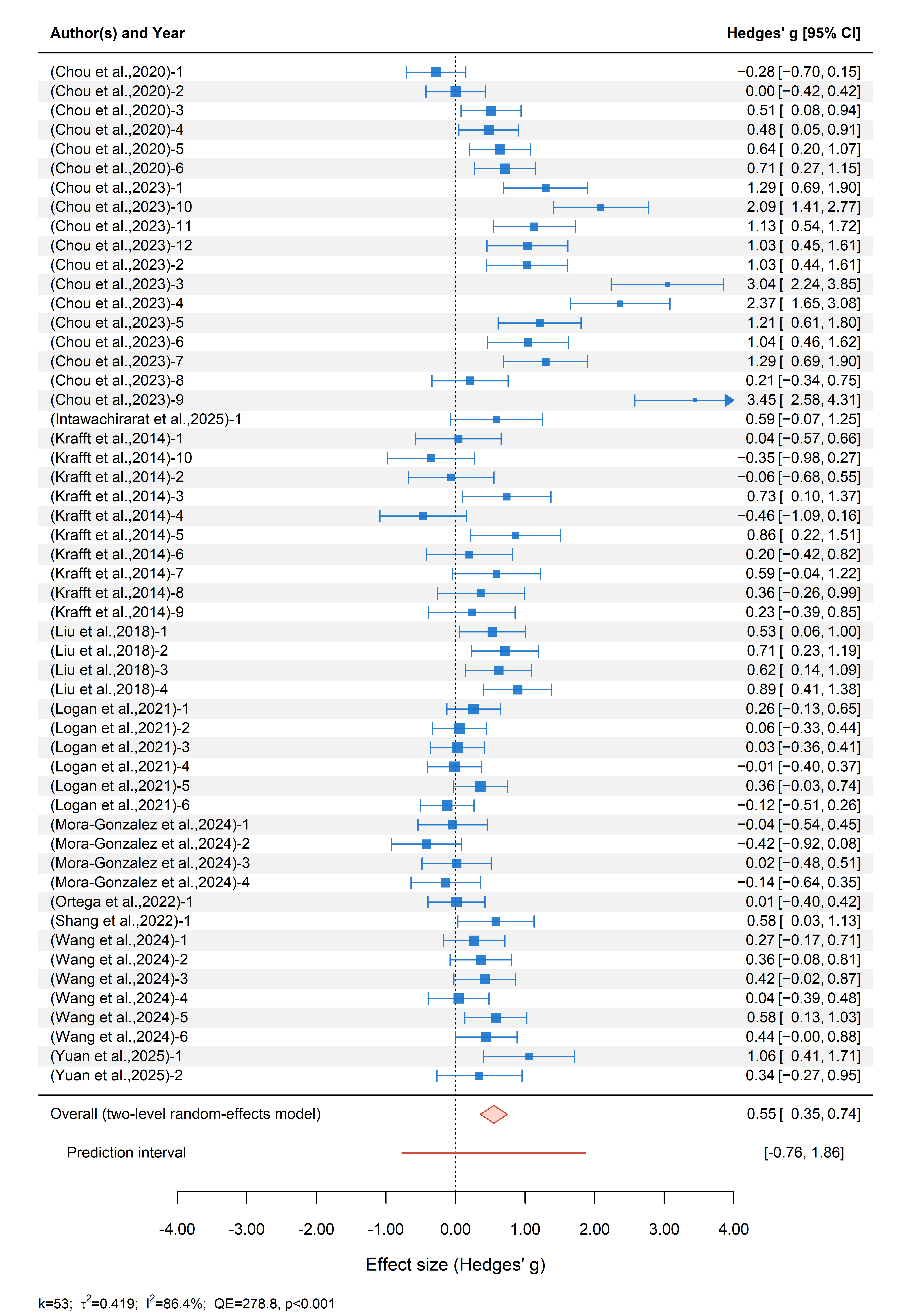


**Fig. S1.** The two-level forest plot of the effect of exercise intervention on inhibitory control (with outliers).

**Figure S2**


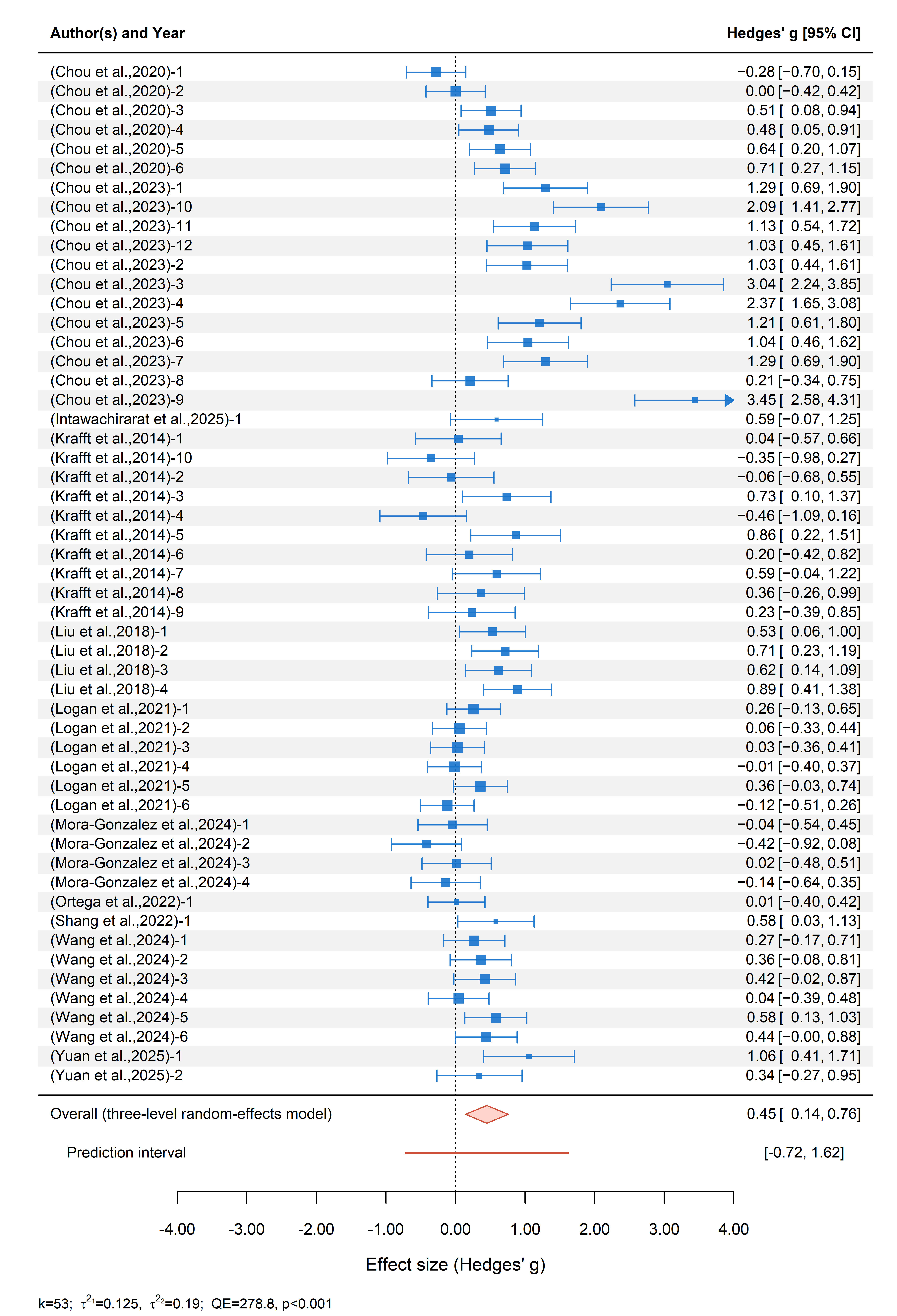


**Fig. S2.** The three-level forest plot of the effect of exercise intervention on inhibitory control (with outliers).

**Figure S3**

**
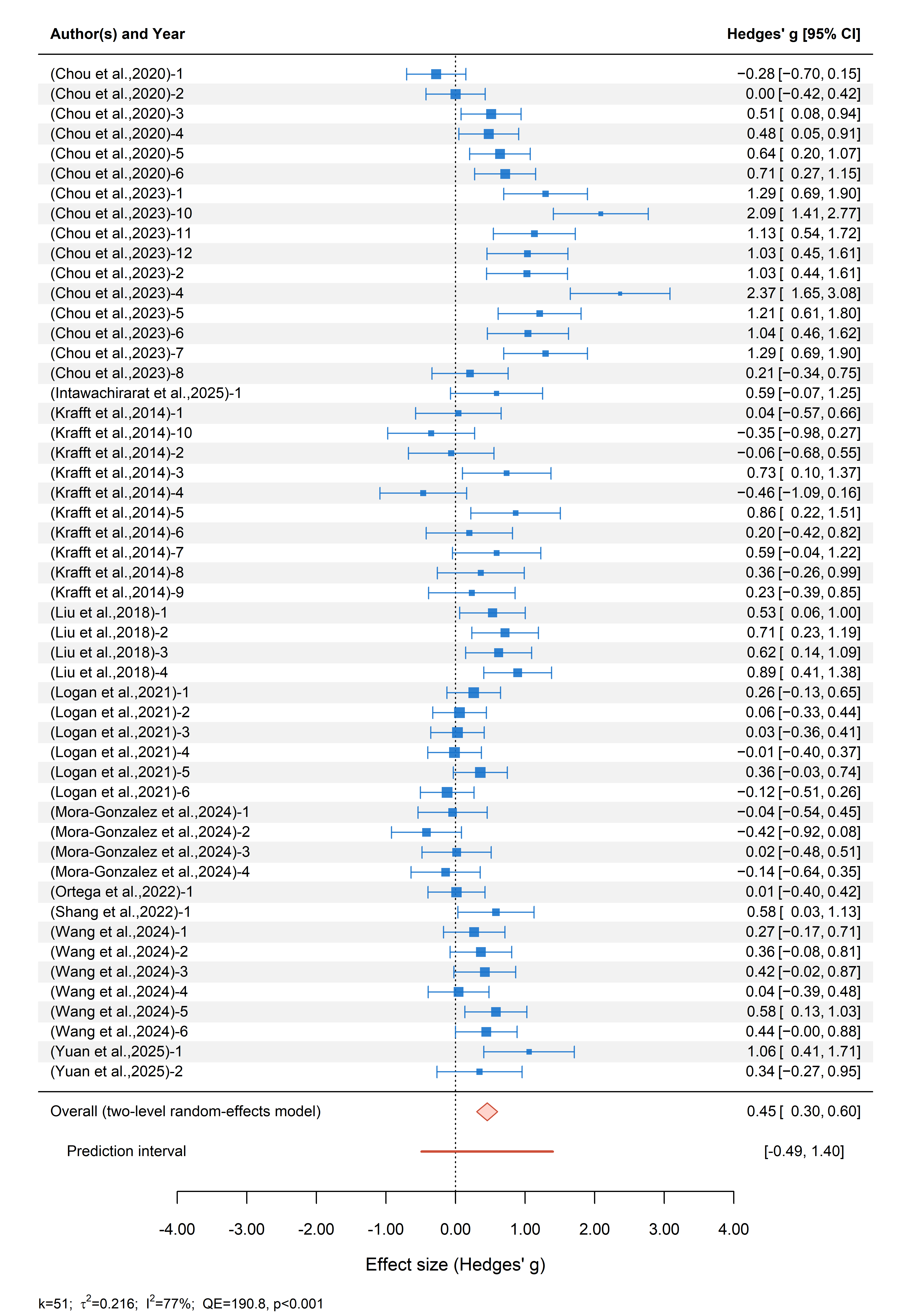
**

**Fig. S3.** The two-level forest plot of the effect of exercise intervention on inhibitory control (no outliers).

**Figure S4**

**
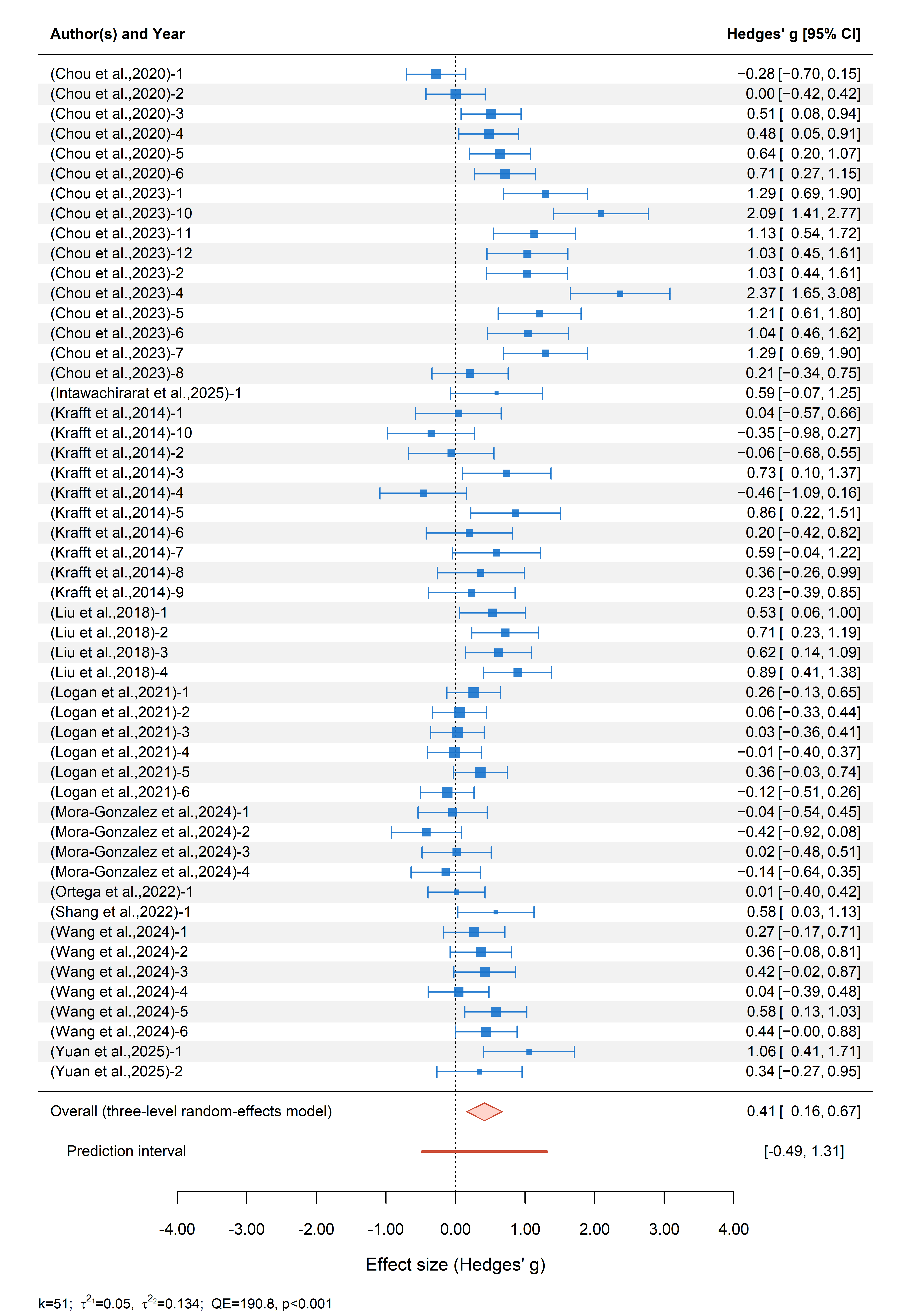
**

**Fig. S4.** The three-level forest plot of the effect of exercise intervention on inhibitory control (no outliers).

### Figure S5


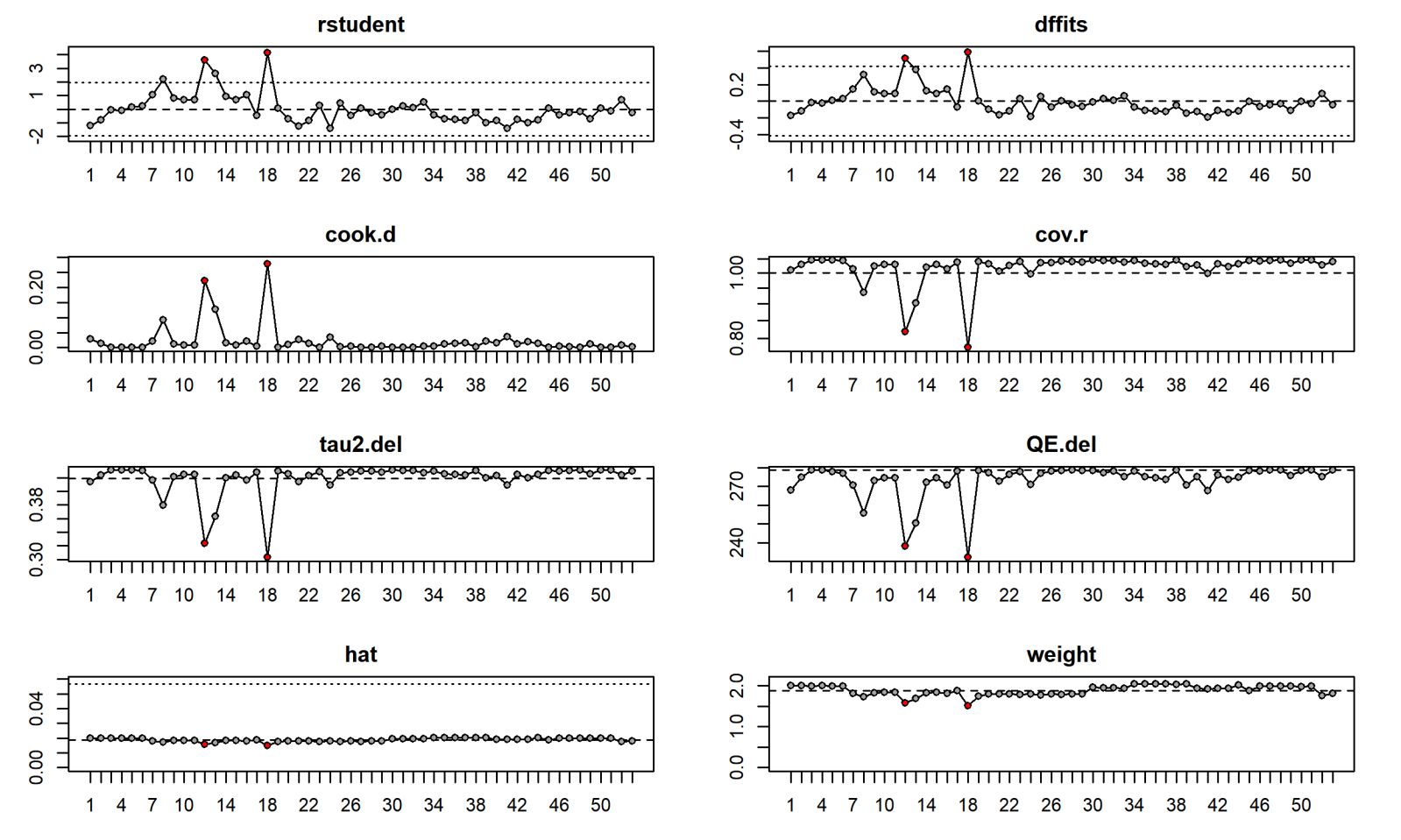


**Fig. S5.** Results of the influence analysis, with influential cases in red.

### Figure S6


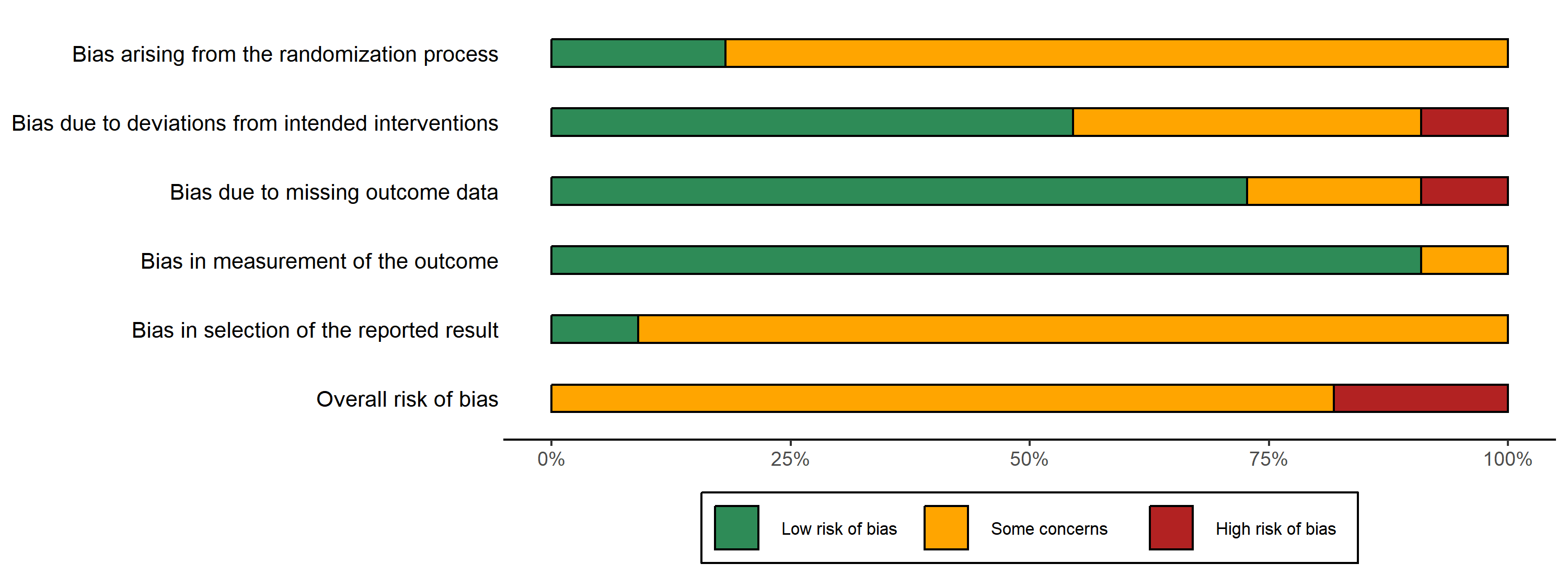


**Fig. S6.** Risk of bias summary of the RoB2 assessments. It was created via robvis (McGuinness & Higgins, 2021)

### Figure S7


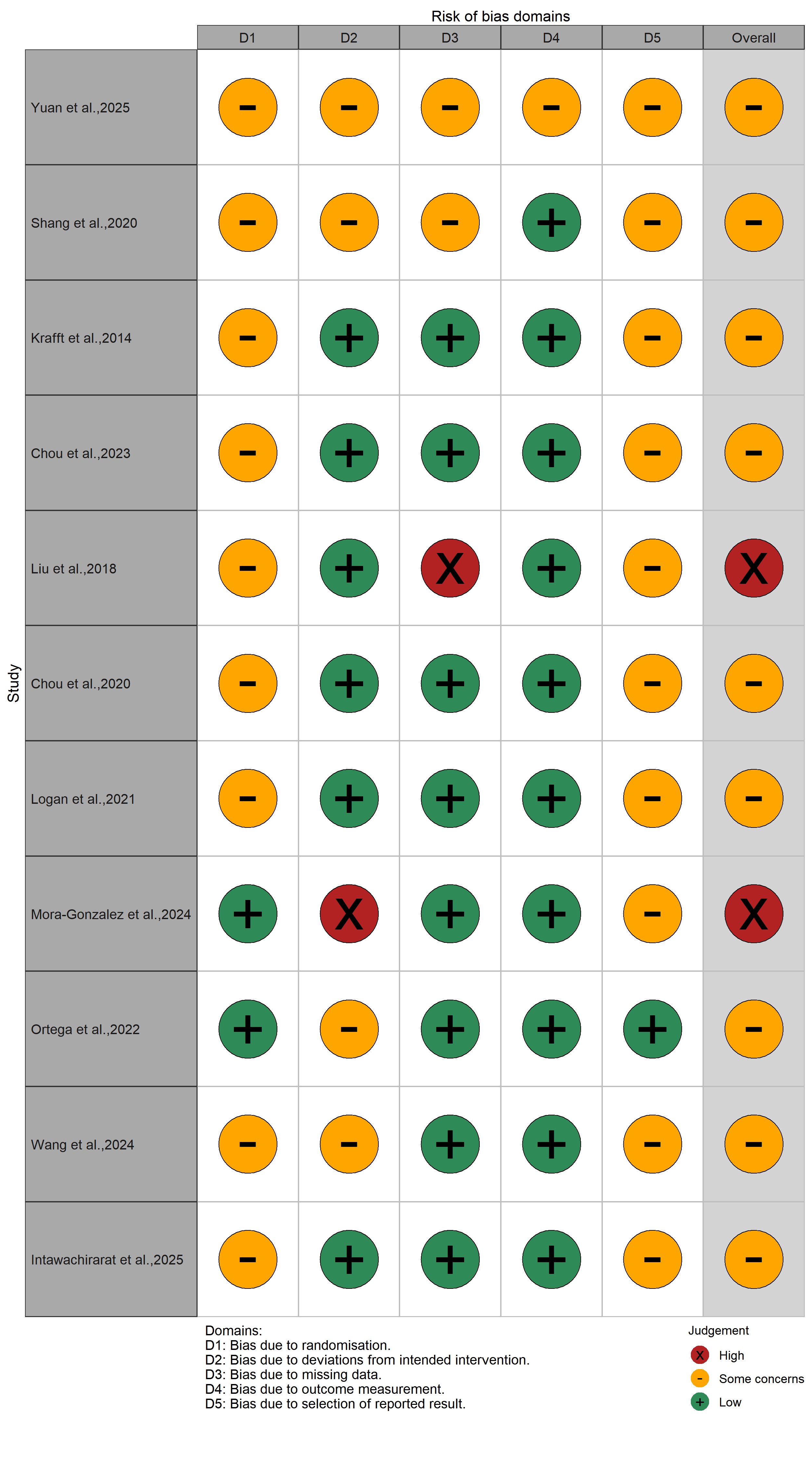


**Fig. S7**. Risk of bias traffic light plot of RoB2 assessments. It was created via robvis (McGuinness & Higgins, 2021).

### Figure S8

###
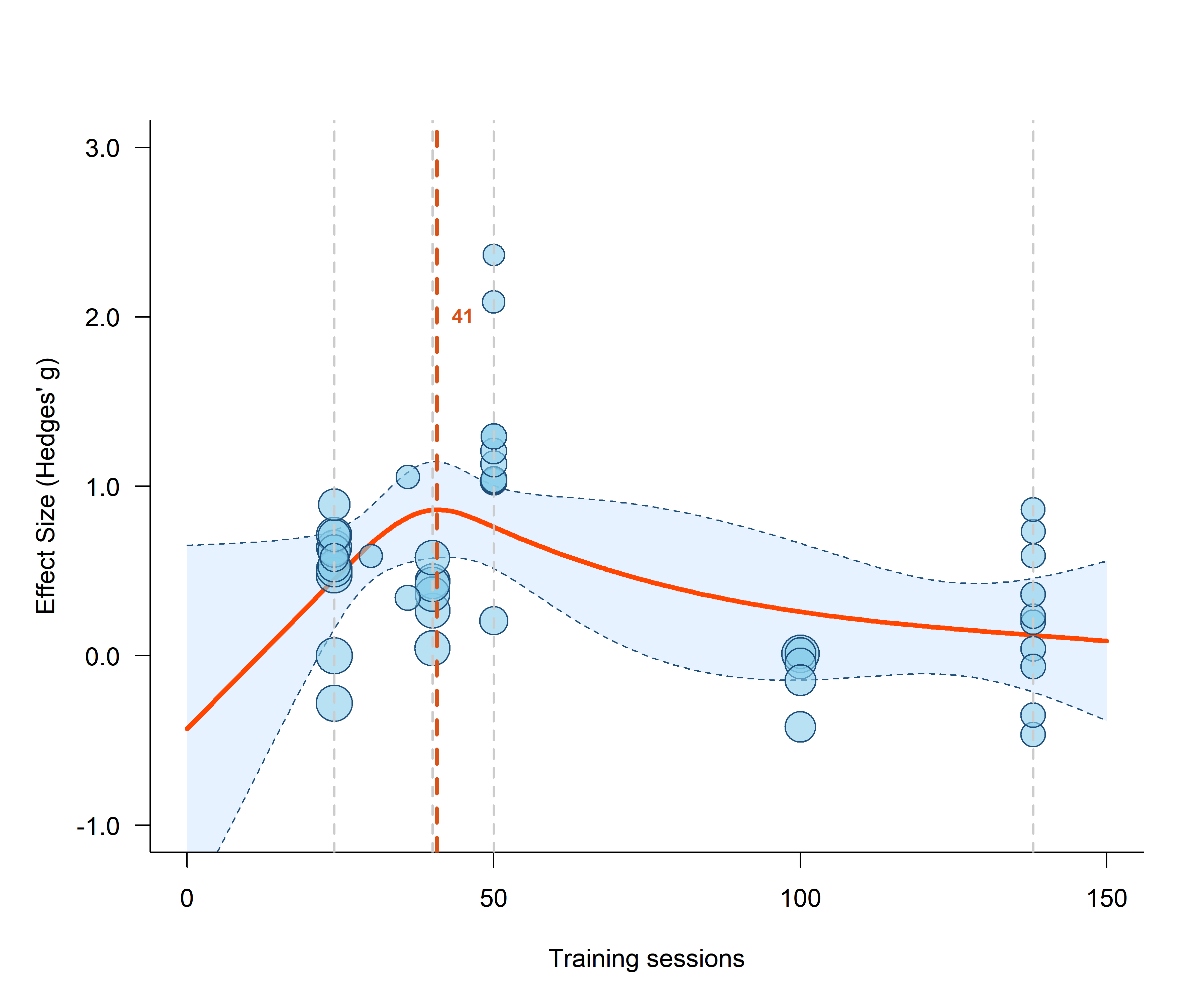


### **Fig. S8**. Effect of training sessions on inhibitory control (after excluding extreme studies).

### Figure S9

###
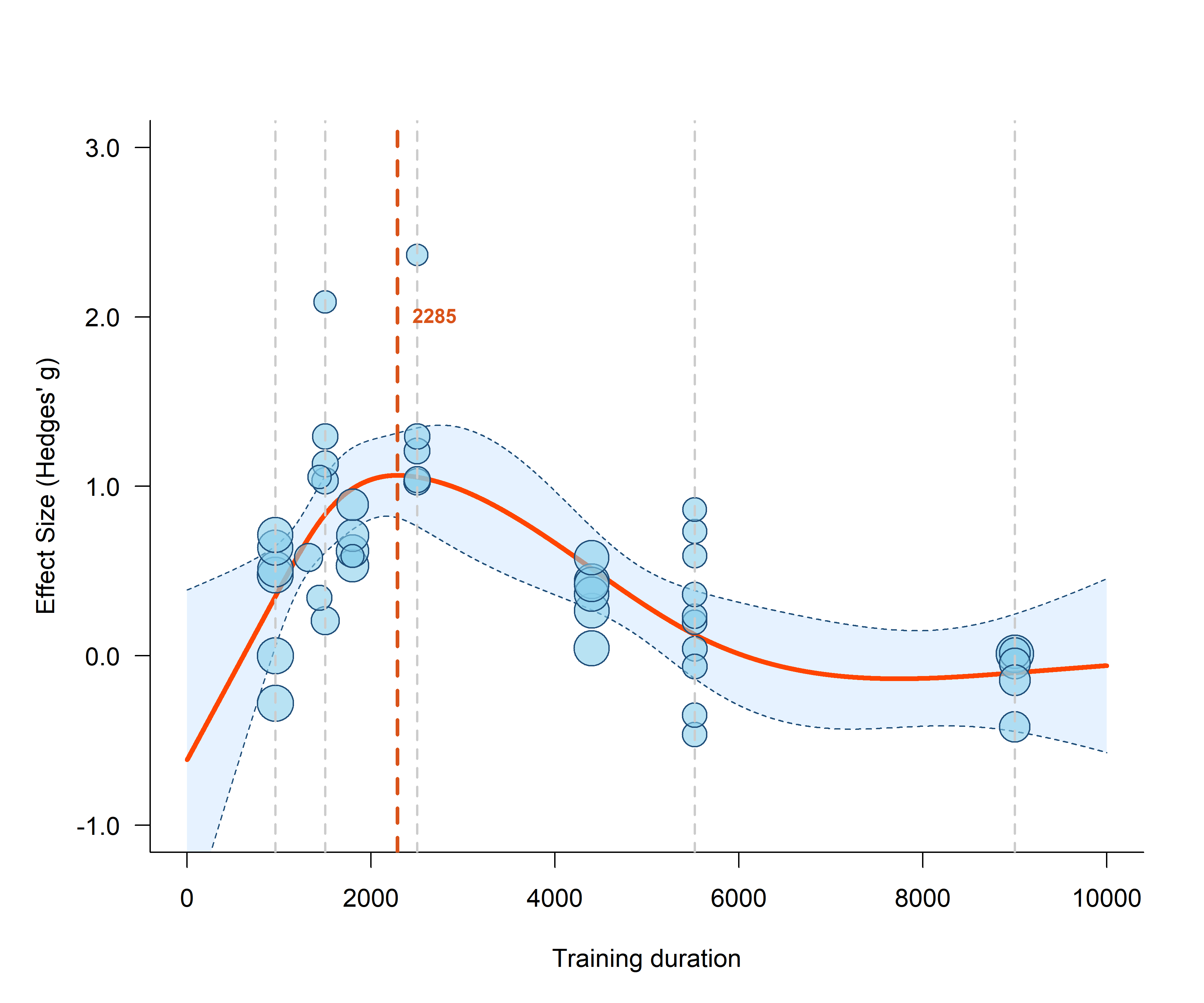


### **Fig. S9**. Effect of training duration on Inhibitory control (after excluding extreme studies).

## 3 PRISMA 2020 Checklist

| **Section and Topic** | **Item #** | **Checklist item** | **Location where item is reported** |
| --- | --- | --- | --- |
| **TITLE** | | |  |
| Title | 1 | Identify the report as a systematic review. | Lines 3-5  (manuscript) |
| **ABSTRACT** | | |  |
| Abstract | 2 | See the PRISMA 2020 for Abstracts checklist. | Lines 26-56  (manuscript) |
| **INTRODUCTION** | | |  |
| Rationale | 3 | Describe the rationale for the review in the context of existing knowledge. | Lines 65-130  (manuscript) |
| Objectives | 4 | Provide an explicit statement of the objective(s) or question(s) the review addresses. | Lines 65-130  (manuscript) |
| **METHODS** | | |  |
| Eligibility criteria | 5 | Specify the inclusion and exclusion criteria for the review and how studies were grouped for the syntheses. | Lines 26-53  (supplementary material) |
| Information sources | 6 | Specify all databases, registers, websites, organisations, reference lists and other sources searched or consulted to identify studies. Specify the date when each source was last searched or consulted. | Lines 138-143  (manuscript) |
| Search strategy | 7 | Present the full search strategies for all databases, registers and websites, including any filters and limits used. | Lines 6-8  (supplementary material) |
| Selection process | 8 | Specify the methods used to decide whether a study met the inclusion criteria of the review, including how many reviewers screened each record and each report retrieved, whether they worked independently, and if applicable, details of automation tools used in the process. | Lines 165-176  (manuscript) |
| Data collection process | 9 | Specify the methods used to collect data from reports, including how many reviewers collected data from each report, whether they worked independently, any processes for obtaining or confirming data from study investigators, and if applicable, details of automation tools used in the process. | Lines 177-186  (manuscript) |
| Data items | 10a | List and define all outcomes for which data were sought. Specify whether all results that were compatible with each outcome domain in each study were sought (e.g. for all measures, time points, analyses), and if not, the methods used to decide which results to collect. | Lines 54-103  (supplementary material) |
|  | 10b | List and define all other variables for which data were sought (e.g. participant and intervention characteristics, funding sources). Describe any assumptions made about any missing or unclear information. | Lines 54-103  (supplementary material) |
| Study risk of bias assessment | 11 | Specify the methods used to assess risk of bias in the included studies, including details of the tool(s) used, how many reviewers assessed each study and whether they worked independently, and if applicable, details of automation tools used in the process. | Lines 202-210  (manuscript) |
| Effect measures | 12 | Specify for each outcome the effect measure(s) (e.g. risk ratio, mean difference) used in the synthesis or presentation of results. | Lines 194-201  (manuscript) |
| Synthesis methods | 13a | Describe the processes used to decide which studies were eligible for each synthesis (e.g. tabulating the study intervention characteristics and comparing against the planned groups for each synthesis (item #5)). | Lines 105-106  (supplementary material) |
|  | 13b | Describe any methods required to prepare the data for presentation or synthesis, such as handling of missing summary statistics, or data conversions. | Lines108-119  (supplementary material) |
|  | 13c | Describe any methods used to tabulate or visually display results of individual studies and syntheses. | Lines121-126  (supplementary material) |
|  | 13d | Describe any methods used to synthesize results and provide a rationale for the choice(s). If meta-analysis was performed, describe the model(s), method(s) to identify the presence and extent of statistical heterogeneity, and software package(s) used. | Lines127-130  (supplementary material) |
|  | 13e | Describe any methods used to explore possible causes of heterogeneity among study results (e.g. subgroup analysis, meta-regression). | Lines131-140  (supplementary material) |
|  | 13f | Describe any sensitivity analyses conducted to assess robustness of the synthesized results. | Lines135-140  (supplementary material) |
| Reporting bias assessment | 14 | Describe any methods used to assess risk of bias due to missing results in a synthesis (arising from reporting biases). | Lines154-158  (supplementary material) |
| Certainty assessment | 15 | Describe any methods used to assess certainty (or confidence) in the body of evidence for an outcome. | Lines212-221  (manuscript) |
| **RESULTS** | | |  |
| Study selection | 16a | Describe the results of the search and selection process, from the number of records identified in the search to the number of studies included in the review, ideally using a flow diagram. | Lines 223-233  (manuscript) |
|  | 16b | Cite studies that might appear to meet the inclusion criteria, but which were excluded, and explain why they were excluded. | Lines 223-233  (manuscript) |
| Study characteristics | 17 | Cite each included study and present its characteristics. | Table S7  (supplementary material) |
| Risk of bias in studies | 18 | Present assessments of risk of bias for each included study. | Lines 355-368  (manuscript) |
| Results of individual studies | 19 | For all outcomes, present, for each study: (a) summary statistics for each group (where appropriate) and (b) an effect estimate and its precision (e.g. confidence/credible interval), ideally using structured tables or plots. | Lines 237-243  (manuscript) |
| Results of syntheses | 20a | For each synthesis, briefly summarise the characteristics and risk of bias among contributing studies. | Lines 226-233  (manuscript) |
|  | 20b | Present results of all statistical syntheses conducted. If meta-analysis was done, present for each the summary estimate and its precision (e.g. confidence/credible interval) and measures of statistical heterogeneity. If comparing groups, describe the direction of the effect. | Lines 237-243  (manuscript) |
|  | 20c | Present results of all investigations of possible causes of heterogeneity among study results. | Lines 266-282  (manuscript) |
|  | 20d | Present results of all sensitivity analyses conducted to assess the robustness of the synthesized results. | Lines 244-255  (manuscript) |
| Reporting biases | 21 | Present assessments of risk of bias due to missing results (arising from reporting biases) for each synthesis assessed. | Lines 355-368  (manuscript) |
| Certainty of evidence | 22 | Present assessments of certainty (or confidence) in the body of evidence for each outcome assessed. | Lines 382-388  (manuscript) |
| **DISCUSSION** | | |  |
| Discussion | 23a | Provide a general interpretation of the results in the context of other evidence. | Lines 389-512  (manuscript) |
|  | 23b | Discuss any limitations of the evidence included in the review. | Lines 514-529  (manuscript) |
|  | 23c | Discuss any limitations of the review processes used. | Lines 514-529  (manuscript) |
|  | 23d | Discuss implications of the results for practice, policy, and future research. | Lines 530-533  (manuscript) |
| **OTHER INFORMATION** | | |  |
| Registration and protocol | 24a | Provide registration information for the review, including register name and registration number, or state that the review was not registered. | Lines 133-136  (manuscript) |
|  | 24b | Indicate where the review protocol can be accessed, or state that a protocol was not prepared. | Lines 133-136  (manuscript) |
|  | 24c | Describe and explain any amendments to information provided at registration or in the protocol. | Lines 133-136  (manuscript) |
| Support | 25 | Describe sources of financial or non-financial support for the review, and the role of the funders or sponsors in the review. | Lines 562-565  (manuscript) |
| Competing interests | 26 | Declare any competing interests of review authors. | Lines 559-560  (manuscript) |
| Availability of data, code and other materials | 27 | Report which of the following are publicly available and where they can be found: template data collection forms; data extracted from included studies; data used for all analyses; analytic code; any other materials used in the review. | Lines 556-558  (manuscript) |

*From: Page MJ, McKenzie JE, Bossuyt PM, Boutron I, Hoffmann TC, Mulrow CD, et al. The PRISMA 2020 statement: an updated guideline for reporting systematic reviews. BMJ 2021;372:n71. doi: 10.1136/bmj.n71. This work is licensed under CC BY 4.0. To view a copy of this license, visit <https://creativecommons.org/licenses/by/4.0/>*

**References**

1. Cole TJ, Bellizzi MC, Flegal KM, Dietz WH: **Establishing a standard definition for child overweight and obesity worldwide: international survey**. *Bmj* 2000, **320**(7244):1240-1243.

2. Zhang L, Chu CH, Liu JH, Chen FT, Nien JT, Zhou C, Chang YK: **Acute coordinative exercise ameliorates general and food-cue related cognitive function in obese adolescents**. *J Sports Sci* 2020, **38**(8):953-960.

3. Zhang L, Wang D, Liu S, Ren FF, Chi L, Xie C: **Effects of Acute High-Intensity Interval Exercise and High-Intensity Continuous Exercise on Inhibitory Function of Overweight and Obese Children**. *Int J Environ Res Public Health* 2022, **19**(16).

4. Sherman EM, Brooks BL: **Behavior rating inventory of executive function–preschool version (BRIEF-P): Test review and clinical guidelines for use**. *Child Neuropsychology* 2010, **16**(5):503-519.

5. Coe BC, Munoz DP: **Mechanisms of saccade suppression revealed in the anti-saccade task**. *Philosophical Transactions of the Royal Society B: Biological Sciences* 2017, **372**(1718):20160192.

6. Kopp B, Rist F, Mattler U: **N200 in the flanker task as a neurobehavioral tool for investigating executive control**. *Psychophysiology* 1996, **33**(3):282-294.

7. Golden C, Freshwater SM, Golden Z: **Stroop color and word test**. 1978.

8. Falkenstein M, Hoormann J, Hohnsbein J: **ERP components in Go/Nogo tasks and their relation to inhibition**. *Acta psychologica* 1999, **101**(2-3):267-291.

9. Best JR, Miller PH: **A developmental perspective on executive function**. *Child development* 2010, **81**(6):1641-1660.

10. Diamond A: **Executive functions**. *Annual review of psychology* 2013, **64**(1):135-168.

11. Fosco WD, Hawk Jr LW, Colder CR, Meisel SN, Lengua LJ: **The development of inhibitory control in adolescence and prospective relations with delinquency**. *Journal of Adolescence* 2019, **76**:37-47.

12. Luna B, Garver KE, Urban TA, Lazar NA, Sweeney JA: **Maturation of cognitive processes from late childhood to adulthood**. *Child development* 2004, **75**(5):1357-1372.

13. Schmidt RA, Lee TD, Winstein C, Wulf G, Zelaznik HN: **Motor control and learning: A behavioral emphasis**: Human kinetics; 2018.

14. Magill R, Anderson DI: **Motor learning and control**: McGraw-Hill Publishing New York; 2010.

15. Shi P, Feng X: **Motor skills and cognitive benefits in children and adolescents: Relationship, mechanism and perspectives**. *Frontiers in Psychology* 2022, **13**:1017825.

16. Bishop DJ, Beck B, Biddle SJ, Denay KL, Ferri A, Gibala MJ, Headley S, Jones AM, Jung M, Lee MJ-C: **TEMPORARY REMOVAL: Physical activity and exercise intensity terminology: a joint American College of Sports Medicine (ACSM) expert statement and exercise and sport science Australia (ESSA) consensus statement**. *Journal of Science and Medicine in Sport* 2024.

17. Ortega FB, Mora-Gonzalez J, Cadenas-Sanchez C, Esteban-Cornejo I, Migueles JH, Solis-Urra P, Verdejo-Román J, Rodriguez-Ayllon M, Molina-Garcia P, Ruiz JR *et al*: **Effects of an Exercise Program on Brain Health Outcomes for Children With Overweight or Obesity: The ActiveBrains Randomized Clinical Trial**. *JAMA Netw Open* 2022, **5**(8):e2227893.

18. Tanaka H, Monahan KD, Seals DR: **Age-predicted maximal heart rate revisited**. *Journal of the american college of cardiology* 2001, **37**(1):153-156.

19. Bravo Oro A, Navarro-Calvillo ME, Esmer C: **Autistic Behavior Checklist (ABC) and its applications**. In: *Comprehensive guide to autism.* edn.: Springer; 2014: 2787-2798.

20. Aksayli ND, Sala G, Gobet F: **The cognitive and academic benefits of Cogmed: A meta-analysis**. *Educational Research Review* 2019, **27**:229-243.

21. Viechtbauer W: **Conducting meta-analyses in R with the metafor package**. *Journal of statistical software* 2010, **36**:1-48.

22. Borenstein M, Hedges LV, Higgins JP, Rothstein HR: **A basic introduction to fixed‐effect and random‐effects models for meta‐analysis**. *Research synthesis methods* 2010, **1**(2):97-111.

23. Morris SB: **Estimating effect sizes from pretest-posttest-control group designs**. *Organizational research methods* 2008, **11**(2):364-386.

24. Schmidt FL, Hunter JE: **Methods of meta-analysis**. *Methods of meta-analysis* 2015.

25. Assink M, Wibbelink CJ: **Fitting three-level meta-analytic models in R: A step-by-step tutorial**. *The Quantitative Methods for Psychology* 2016, **12**(3):154-174.

26. Cheung MW-L: **Modeling dependent effect sizes with three-level meta-analyses: a structural equation modeling approach**. *Psychological methods* 2014, **19**(2):211.

27. Cheung MW-L: **A guide to conducting a meta-analysis with non-independent effect sizes**. *Neuropsychology review* 2019, **29**(4):387-396.

28. Melby-Lervåg M, Redick TS, Hulme C: **Working memory training does not improve performance on measures of intelligence or other measures of “far transfer” evidence from a meta-analytic review**. *Perspectives on Psychological Science* 2016, **11**(4):512-534.

29. Rao SJ: **Regression modeling strategies: with applications to linear models, logistic regression, and survival analysis**. In*.*: Taylor & Francis; 2003.

30. Harrer M, Cuijpers P, Furukawa T, Ebert D: **Doing meta-analysis with R: A hands-on guide**: Chapman and Hall/CRC; 2021.

31. Egger M, Smith GD, Schneider M, Minder C: **Bias in meta-analysis detected by a simple, graphical test**. *bmj* 1997, **315**(7109):629-634.

32. Duval S, Tweedie R: **A nonparametric “trim and fill” method of accounting for publication bias in meta-analysis**. *Journal of the american statistical association* 2000, **95**(449):89-98.

33. Rodgers MA, Pustejovsky JE: **Evaluating meta-analytic methods to detect selective reporting in the presence of dependent effect sizes**. *Psychological methods* 2021, **26**(2):141.
